# Suppression of the Nrf2-Dependent Antioxidant Response by Glucocorticoids and 11β-HSD1-Mediated Glucocorticoid Activation in Hepatic Cells

**DOI:** 10.1371/journal.pone.0036774

**Published:** 2012-05-11

**Authors:** Denise V. Kratschmar, Diego Calabrese, Jo Walsh, Adam Lister, Julia Birk, Christian Appenzeller-Herzog, Pierre Moulin, Chris E. Goldring, Alex Odermatt

**Affiliations:** 1 Swiss Center for Applied Human Toxicology and Division of Molecular and Systems Toxicology, Department of Pharmaceutical Sciences, University of Basel, Basel, Switzerland; 2 Novartis Institute for Biomedical Research – PCS – iTox – Molecular Pathology and Immunology, Novartis Pharma AG, Basel, Switzerland; 3 MRC Centre for Drug Safety Science, Department of Clinical and Molecular Pharmacology, Institute of Translational Medicine, University of Liverpool, Liverpool, United Kingdom; University of Texas Health Science Center at San Antonio/Greehey CCRI, United States of America

## Abstract

**Background:**

Nuclear factor (erythroid-derived 2)-like 2 (Nrf2) is a key transcription factor regulating a plethora of detoxifying enzymes and antioxidant genes involved in drug metabolism and defence against oxidative stress. The glucocorticoid receptor (GR) is a ligand-induced transcription factor involved in the regulation of energy supply for metabolic needs to cope with various stressors. GR activity is controlled by glucocorticoids, which are synthesized in the adrenal glands and regenerated mainly in the liver from inactive cortisone by 11β-hydroxysteroid dehydrogenase-1 (11β-HSD1).

**Methods and Principal Findings:**

Using transfected HEK-293 cells and hepatic H4IIE cells we show that glucocorticoids, activated by 11β-HSD1 and acting through GR, suppress the Nrf2-dependent antioxidant response. The expression of the marker genes *NQO1*, *HMOX1* and *GST2A* was suppressed upon treatment of 11β-HSD1 expressing cells with cortisone, an effect that was reversed by 11β-HSD1 inhibitors. Furthermore, our results demonstrate that elevated glucocorticoids lowered the ability of cells to detoxify H_2_O_2_. Moreover, a comparison of gene expression in male and female rats revealed an opposite sexual dimorphism with an inverse relationship between 11β-HSD1 and Nrf2 target gene expression.

**Conclusions:**

The results demonstrate a suppression of the cellular antioxidant defence capacity by glucocorticoids and suggest that elevated 11β-HSD1 activity may lead to impaired Nrf2-dependent antioxidant response. The gender-specific differences in hepatic expression levels of 11β-HSD1 and Nrf2 target genes and the impact of pharmacological inhibition of 11β-HSD1 on improving cellular capacity to cope with oxidative stress warrants further studies *in vivo*.

## Introduction

The liver is metabolically highly active, essentially regulating carbohydrate and lipid metabolism as well as the detoxification of xenobiotics and reactive endogenous chemicals. Various cytoprotective genes, including those under the control of the transcription factor nuclear factor (erythroid 2)-like 2 (Nrf2, *NFE2L2* gene), are expressed in hepatocytes to avoid cellular damage by reactive compounds. Nrf2 is the key player of the tightly regulated antioxidant cell defense system [1]. Upon recognition of the antioxidant responsive elements (ARE) on the promoters of its target genes, Nrf2 modulates basal and ligand-induced expression of various cytoprotective enzymes [2]. The importance of Nrf2 is shown in knockout mice, exhibiting an enhanced susceptibility towards oxidative stress caused by xenobiotics due to diminished expression of cytoprotective genes [3], [4], [5], [6]. Nrf2 target genes include essential phase II detoxification enzymes such as NAD(P)H:quinone oxidoreductases (NQO) [7], heme oxygenase-1 (HO-1, *HMOX1* gene) [8] and glutathione S-transferases (GST) [1], [9] that are induced by oxidative stress caused by xenobiotics, antioxidants, UV-light, and ionizing radiation [2].

A recent study reported gender-divergent expression of NQO1 in specific rat strains studied [10]. Hepatic basal NQO1 mRNA expression was two-fold lower in male compared with female Sprague Dawley rats. Induction of NQO1 expression with the classical Nrf2 inducers butylated hydroxyanisole and oltipraz was more pronounced in female compared with male rats. Importantly, it has been reported that male rats have higher susceptibility to carcinogenic xenobiotics [11]. Interestingly, gender-related differences were also found for humans [12]; however, the underlying mechanisms remain unknown.

Decreased Nrf2-mediated constitutive and oltipraz- or tert-butylhydroquinone (t-BHQ)-inducible *GSTA2* gene expression was found in rat H4IIE hepatoma cells upon activation of the glucocorticoid receptor (GR) by dexamethasone [13]. Importantly, the oxidized metabolite of dexamethasone, 11-ketodexamethasone, is also a potent GR agonist; thereby dexamethasone circumvents the 11β-hydroxysteroid dehydrogenase (11β-HSD) mediated control of GR activation [14]. Under physiological conditions, hepatic GR function depends on the circulating concentration of glucocorticoids produced by the adrenal glands and on the activity of hepatic 11β-HSD1, which converts the inactive 11-ketoglucocorticoids cortisone and 11-dehydrocorticosterone into their active 11β-hydroxyls cortisol and corticosterone [15]. In mice, the transgenic over expression of 11β-HSD1 specifically in the liver resulted in the development of impaired insulin sensitivity and steatosis, demonstrating the adverse metabolic effects of elevated hepatic glucocorticoid activation [16].

The impact of endogenous glucocorticoids and of 11β-HSD1 on the antioxidant redox pathway has not yet been studied. Therefore, we used rat H4IIE cells, known to express functional Nrf2 and down-stream regulated enzymes [13], [17], [18], and H4IIE cells transiently or stably transfected with 11β-HSD1 to elucidate its impact on the antioxidant response pathway. Moreover, we studied whether the observed gender differences in hepatic 11β-HSD1 expression in rats [19], [20] may correlate with differences in the expression of Nrf2 target genes.

## Results

### Glucocorticoid-mediated inhibition of Nrf2-dependent transactivation in HEK-293 cells

**Figure 1:**
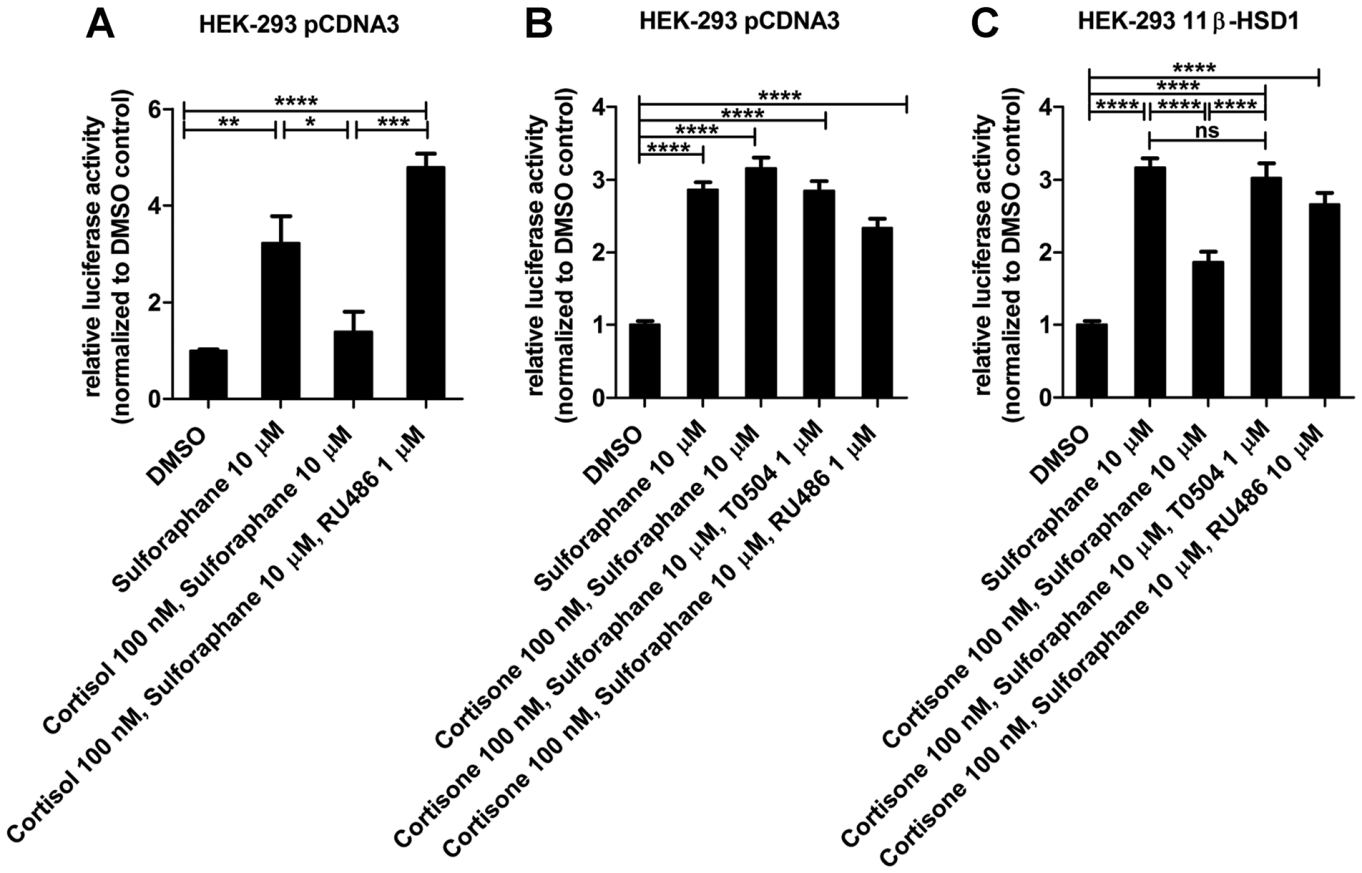
Suppression of Nrf2-dependent reporter gene activation by glucocorticoids in HEK-293 cells. HEK-293 cells transiently transfected with plasmids for Nrf2, GR, ARE8L-reporter, pCMV-LacZ and either pcDNA3 (*A, B*) or 11β-HSD1 (*C*) were incubated for 24 h with vehicle (DMSO), 10 µM sulforaphane, 100 nM cortisone or cortisol, in the presence or absence of 1 µM T0504 or RU-486. Data (mean ± SD) were obtained from three independent experiments each measured in triplicate. *, *p*<0.05, **, *p*<0.01, ***, *p*<0.001, *p*-value was obtained using one-way ANOVA followed by Bonferroni post-tests compared with vehicle control (DMSO).

To assess whether glucocorticoids inhibit Nrf2 function, we transiently expressed Nrf2 and GR together with the ARE8L-luciferase reporter [21] in HEK-293 cells (Fig. 1). Incubation of the cells with 10 µM sulforaphane for 24 h resulted in three- to four-fold increased ARE8L-reporter activity. Activation of GR by simultaneous incubation with the active glucocorticoid cortisol within physiological concentrations (100 nM) for 24 h suppressed Nrf2-dependent transactivation (Fig. 1A). Cortisone, the physiologically inactive form, requires prior activation to cortisol by an enzymatic tissue-specific process catalyzed by 11ß-HSD1. Cortisone in the absence of 11β-HSD1 did not affect sulforaphane-induced reporter gene activation (Fig. 1B) but suppressed reporter gene activation in cells expressing 11β-HSD1, an effect that was fully reversed by the selective 11β-HSD1 inhibitor T0504 (Fig. 1C). The GR antagonist RU-486 also fully restored Nrf2-mediated transactivation. No inhibition of recombinant human 11β-HSD1 (measured using cell lysates) was observed at 2 µM; at 20 µM a weak inhibition with 69±8% remaining activity was obtained (data not shown).

### Induction of the Nrf2-dependent ARE8L-reporter in rat H4IIE hepatoma cells

**Figure 2:**
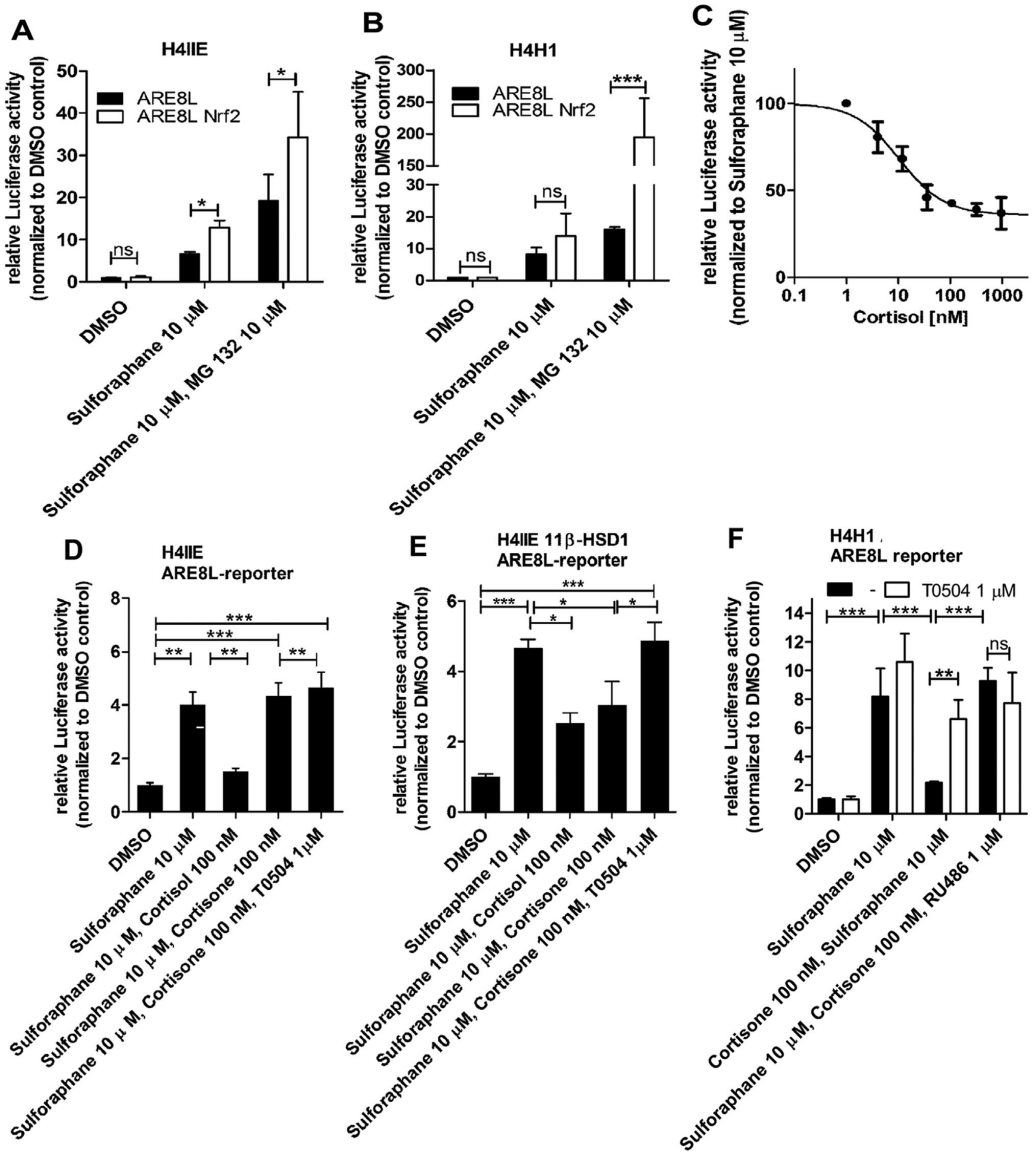
Suppression of Nrf2 transactivation by 11β-HSD1-mediated glucocorticoid activation in H4IIE cells. The activation of the Nrf2-dependent ARE8L-reporter by 10 µM sulforaphane was measured in rat H4IIE hepatoma cells (*A*) and in H4IIE cells stably expressing 11β-HSD1 (H4H1 clone) (*B*) at endogenous Nrf2 expression (black bars) and upon over expression of Nrf2 (white bars). CMV-LacZ plasmid served as a transfection control to normalize luciferase values. Cells were treated with vehicle (DMSO), sulforaphane (10 µM), or sulforaphane and proteasome inhibitor MG132 (10 µM) for 24 h. H4IIE cells transfected with ARE8L-reporter and pCMV-LacZ cells were incubated with sulforaphane and increasing concentrations of glucocorticoids for 24 h, followed by measuring luciferase activity to estimate the effective concentration of cortisol leading to a 50% reduction of the reporter activity (*C*). Suppression of Nrf2 transactivation by glucocorticoids was further studied in H4IIE cells transiently transfected with ARE8L-reporter and pcDNA3 (*D*) or with ARE8L-reporter and 11β-HSD1 (*E*). Cells were incubated with vehicle or sulforaphane, glucocorticoids and vehicle or 11β-HSD1 inhibitor T0504 at the concentrations indicated for 24 h, followed by measuring luciferase activity. The impact of 11β-HSD1 inhibitors and GR antagonists on Nrf2-dependent transactivation was similarly assessed in H4H1 cells transfected with ARE8L-reporter and pCMV-LacZ (*F*). Data represent mean ± SD from at least two independent experiments performed in triplicate. *, p<0.05, **, p<0.01, ***, p<0.001, p-value was obtained using one-way ANOVA followed by Bonferroni post-tests compared with control (DMSO), ns, not significant.

Next, we characterized the responsiveness of the Nrf2 pathway in H4IIE cells transiently transfected with the ARE8L-reporter. As shown in Figure 2A, 10 µM sulforaphane stimulated ARE8L-reporter activity approximately seven-fold. Co-transfection of the cells with recombinant Nrf2 further stimulated ARE8L-reporter activity almost two-fold. It was reported that Nrf2 protein has a short half-life (of about 15 min), which is significantly enhanced by proteasome inhibitors [22]. Upon incubation of H4IIE cells with the proteasome inhibitor MG132 we observed approximately two-fold increased protein levels of Nrf2 and its target NQO1 (Fig. S1). We then assessed ARE8L-reporter activity in cells treated with sulforaphane and the proteasome inhibitor MG132 (10 µM). Total luciferase reporter activity was two to three times higher in the presence of the proteasome inhibitor.

Currently, there are no hepatocellular lines available that express substantial levels of 11β-HSD1. We found that the rat hepatoma HPCT-1E3 cell line [23] expresses low to moderate levels of endogenous 11β-HSD1. However, HPCT-1E3 cells need to be cultivated in the presence of 1.5 µM of the potent glucocorticoid dexamethasone, which makes it a model with limited use to study interactions between glucocorticoids and antioxidant redox pathway. Moreover, although primary hepatocytes express high levels of 11β-HSD1, expression levels rapidly decline upon cultivation, resulting in large inter-experimental differences. Therefore, we recently constructed a H4IIE cell clone stably expressing 11β-HSD1 (designated as H4H1) [24]. The 11β-HSD1 cortisone reductase activity of H4H1 cells is comparable with that of freshly isolated primary hepatocytes but more than ten times higher than that of HPCT-1E3 cells. The Nrf2-dependent ARE8L-reporter was activated in H4H1 cells by sulforaphane both in the presence or absence of co-transfected recombinant Nrf2 (Fig. 2B).

### 11β-HSD1-mediated glucocorticoid activation suppresses Nrf2 transactivation capacity

The observation that over expression and activation of GR in HEK-293 cells inhibits Nrf2-dependent transactivation of the ARE8L-reporter led us to investigate the impact of 11β-HSD1 and glucocorticoids on Nrf2 transactivation in cells expressing endogenous Nrf2 levels. In H4IIE cells expressing endogenous Nrf2, the effective concentration of cortisol leading to a 50% down-regulation of the sulforaphane-induced Nrf2 transactivation capacity was determined to be 10±5 nM (Fig. 2C). In further experiments, incubation of H4IIE cells with 100 nM cortisol almost completely abolished sulforaphane-induced ARE8L-reporter activation. Neither cortisone nor T0504 affected Nrf2-dependent transactivation (Fig. 2D). H4IIE cells are devoid of endogenous 11β-HSD1 expression, as measured by real-time RT-PCR (not shown); therefore, we transiently transfected H4IIE cells with rat 11β-HSD1 and found diminished Nrf2 activity upon addition of cortisol or cortisone (Fig. 2E).

### 11β-HSD1 inhibitors and GR antagonists reverse glucocorticoid-mediated suppression of Nrf2 transactivation

Suppression of Nrf2 transactivation by cortisone in 11β-HSD1 expressing H4IIE cells was fully reversed by 1 µM of the selective 11β-HSD1 inhibitor T0504 (Fig. 2E). To ensure that the observed effects are not due to sulforaphane-dependent 11β-HSD1 inhibition, we measured 11β-HSD1-mediated conversion of cortisone to cortisol in H4H1 cells. Sulforaphane did not affect 11β-HSD1 enzyme activity (Fig. S2). To overcome experimental differences due to transfection efficiency, we studied the impact of 11β-HSD1 inhibition on Nrf2-dependent transactivation in H4H1 cells (Fig. 2F). Incubation with 10 µM sulforaphane for 24 h resulted in an eight-fold activation of the ARE8L-reporter. ARE8L-reporter activity in H4H1 cells treated with sulforaphane was not significantly altered by the addition of T0504. Importantly, following 24 h incubation of H4H1 cells with 100 nM cortisone and 10 µM sulforaphane, Nrf2-dependent activation of the ARE8L-reporter was significantly decreased, and reporter activity was indistinguishable from that of DMSO treated cells. The suppression of Nrf2 function due to 11β-HSD1 activity was almost fully reversed by the 11β-HSD1 inhibitor T0504 or the GR antagonist RU-486.

### Oxidative stress induced by H_2_O_2_ enhances Nrf2-dependent pathway but not 11β-HSD1 expression and GR activity

**Figure 3:**
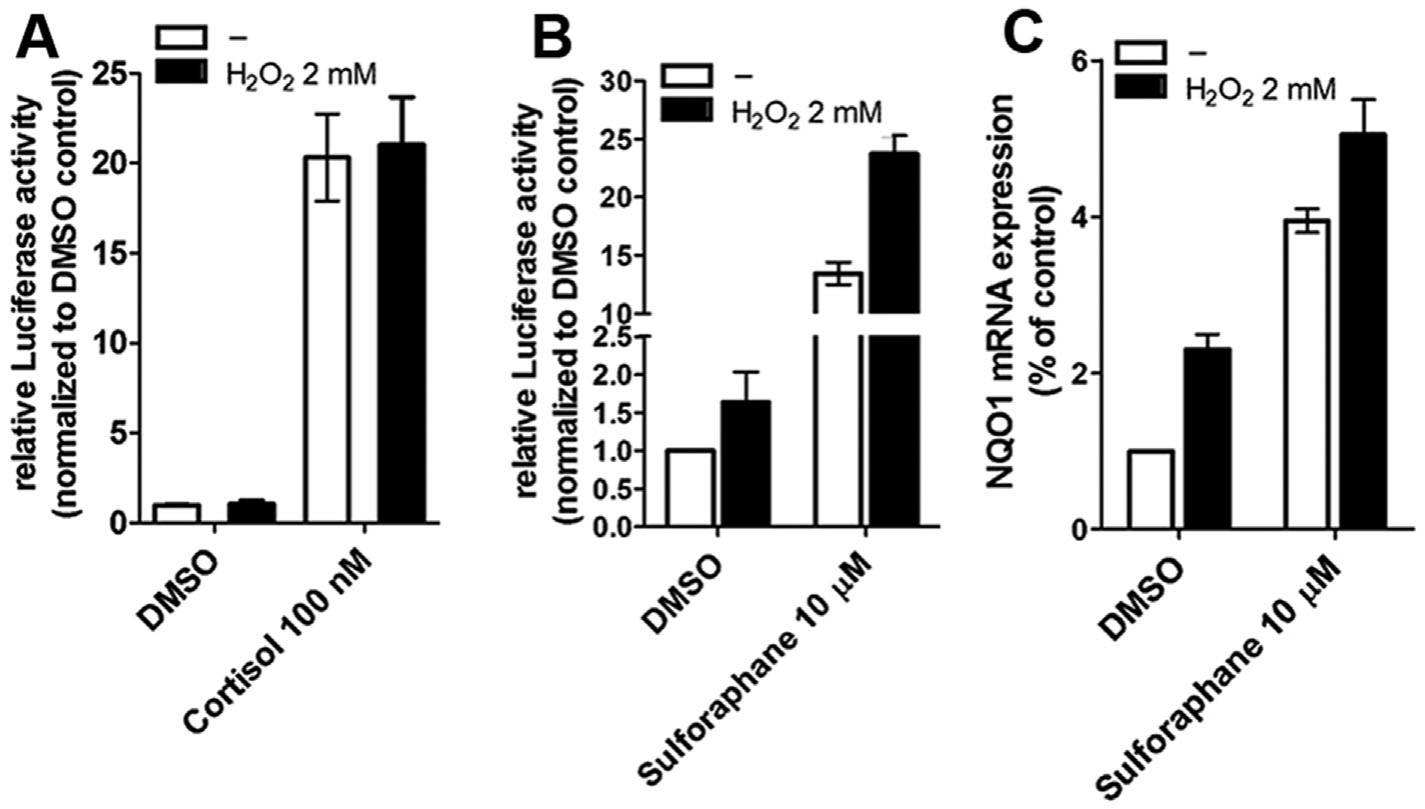
Influence of oxidative stress on Nrf2 pathway and GR transactivation. The activation of the GR-dependent TAT3-TATA-reporter by 100 µM cortisol was measured in rat H4IIE hepatoma cells with endogenous GR expression (*A*). CMV-LacZ plasmid served as transfection control to normalize luciferase values. Cells were treated with vehicle (DMSO) or cortisol (100 µM), with or without H_2_O_2_ (2 mM) for 24 h at 37°C. Data represent mean ± SD from at least three independent experiments performed in triplicate. The influence of oxidative stress induced by H_2_O_2_ on Nrf2-dependent transactivation was measured in ARECS3 cells stably expressing the ARE8L-reporter (*B*). Cells were treated with vehicle (DMSO) or sulforaphane (10 µM) in the presence or absence of H_2_O_2_ (2 mM) for 24 h. Data represent mean ± SD from three independent experiments measured in triplicate. Activation of NQO1 mRNA expression by H_2_O_2_ was measured in H4IIE cells (*C*). Cells were incubated for 24 h at 37°C with vehicle (0.05% DMSO) or sulforaphane (10 µM) in the presence or absence of H_2_O_2_ (2 mM), followed by determination of NQO1 mRNA levels by real-time RT-PCR. Data (mean ± SD from two independent experiments measured in triplicate) represent ratios of NQO1 mRNA to GAPDH control mRNA from treated cells normalized to the values obtained from cells incubated with vehicle (DMSO).

We tested whether H_2_O_2_ might affect 11β-HSD1 activity or GR-dependent transactivation. H_2_O_2_ did not affect 11β-HSD1 activity and GR transactivation at subcytotoxic concentrations (up to 5 µM) in HEK-293 cells (data not shown). H4IIE, H4H1 and HPCT-1E3 cells have a much higher ability to cope with H_2_O_2_ and concentrations up to 2 mM did not cause overt cytotoxic effects. Incubation of H4H1 and HPCT-1E3 cells for 24 h in the presence of 2 mM H_2_O_2_ did not alter 11β-HSD1 mRNA expression and cortisone reductase activity (not shown and Fig. S3). To assess whether H_2_O_2_ influences the GR pathway we assessed the transcriptional activity of the GR by employing a TAT3-TATA reporter gene in H4IIE cells (Fig. 3A). Cells were incubated with cortisol (100 nM) with or without H_2_O_2_ (2 mM) for 24 h. Cortisol treatment resulted in 20-fold increased TAT3-TATA-reporter activity and the reporter response was indistinguishable from cells treated simultaneously with cortisol and H_2_O_2_.

In contrast, oxidative stress increased Nrf2-dependent antioxidant response. We investigated whether H_2_O_2_ induces ARE8L reporter activity in H4IIE cells stably expressing this reporter (designated as ARECS3 clone). ARECS3 cells were treated with vehicle (0.05% DMSO) or sulforaphane (10 µM) in the presence or absence of H_2_O_2_ (2 mM). H_2_O_2_-induced oxidative stress increased both basal as well as sulforaphane-induced activity of the ARE8L reporter (Fig. 3B). In addition, H_2_O_2_ enhanced basal as well as sulforaphane-induced NQO1 mRNA in H4IIE cells treated with vehicle (0.05% DMSO) or sulforaphane (10 µM) (Fig. 3C).

### Downregulation of NQO1 and GST2A mRNA expression by cortisol in H4IIE cells

**Figure 4:**
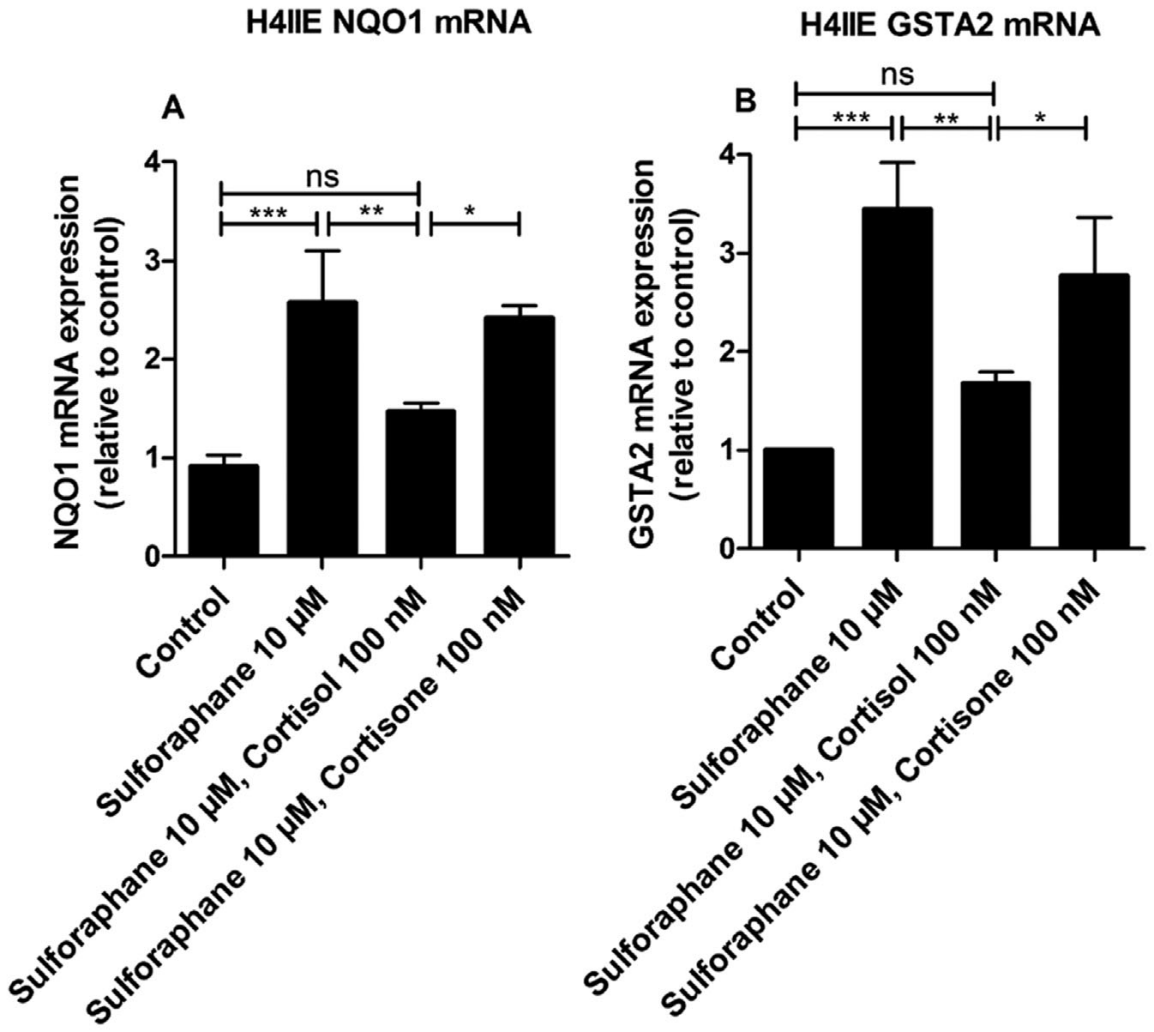
Inhibition of Nrf2-induced mRNA expression of NQO1 and GSTA2 by cortisol. H4IIE cells were incubated for 24 h at 37°C with 10 µM sulforaphane in the absence or presence of 100 nM cortisol or cortisone, respectively, followed by determination of NQO1 (A) and GSTA2 mRNA levels (B) by real-time RT-PCR. Data (mean ± S.D. from three independent experiments performed in triplicate) represent ratios of NQO1 and GSTA2 mRNA to GAPDH control mRNA from treated cells normalized to the values obtained from cells incubated with vehicle (DMSO). *, *p*<0.05, **, *p*<0.01, ***, *p*<0.001, *p*-values were obtained using one-way ANOVA followed by Bonferroni post-tests compared with vehicle control (DMSO).

Next, we determined the expression of NQO1 and GSTA2 mRNA in H4IIE cells treated with sulforaphane in the absence or presence of glucocorticoids. Treatment with sulforaphane enhanced NQO1 and GSTA2 mRNA expression approximately 2.5- and 3.5-fold, respectively, compared with DMSO treated controls. Co-incubation of H4IIE cells with sulforaphane and 100 nM cortisol resulted in significantly lower NQO1 and GSTA2 mRNA expression, whereas cortisone in the absence of 11β-HSD1 was ineffective (Fig. 4).

### Cortisol suppresses NQO1 and Nrf2 protein levels induced by H_2_O_2_ in H4IIE cells

**Figure 5:**
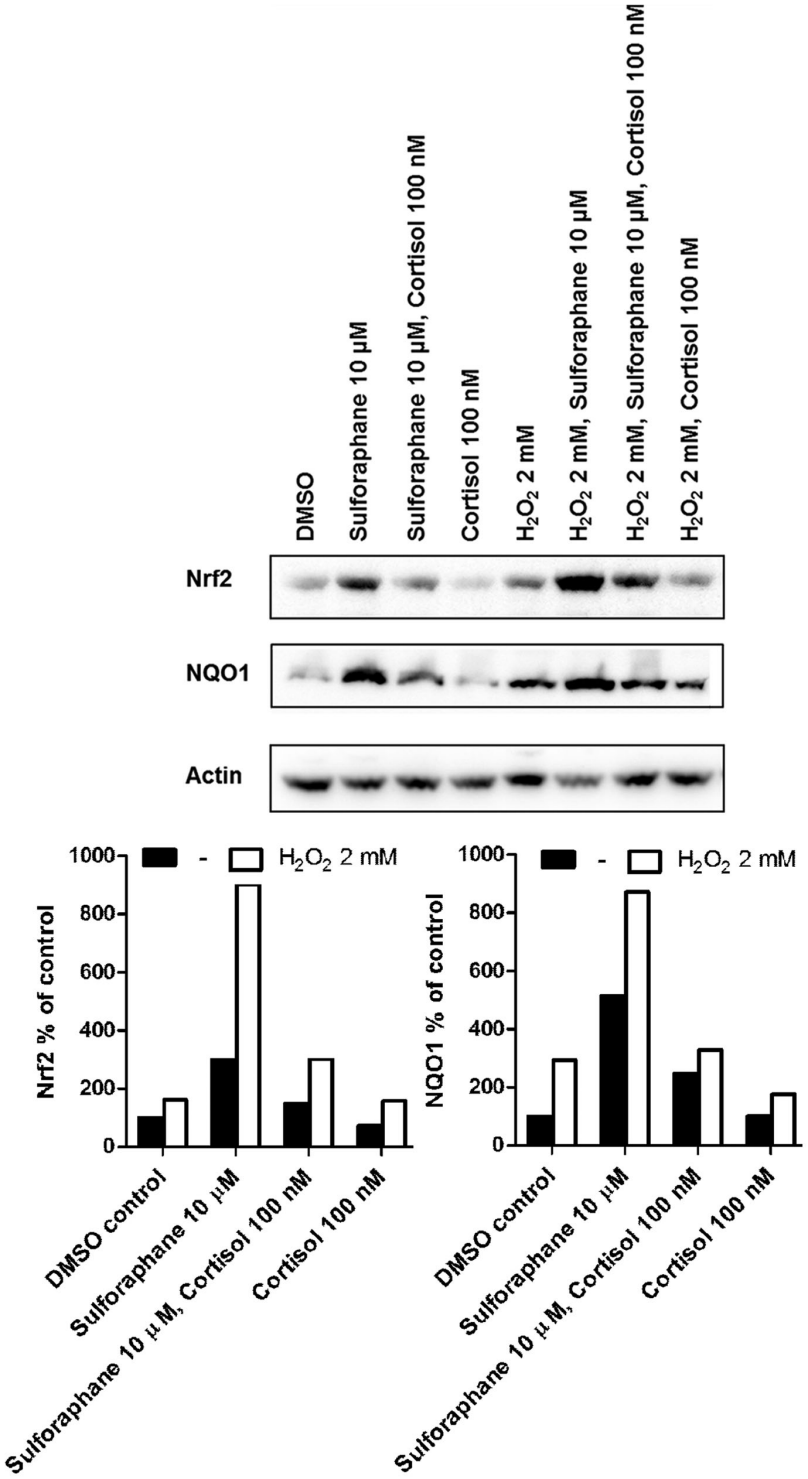
Suppression of Nrf2 and NQO1 protein expression by cortisol in oxidative stress-induced H4IIE cells. H4IIE cells were treated for 24 h with vehicle (DMSO), cortisone, sulforaphane or cortisone and sulforaphane in the presence or absence of H_2_O_2_. Cells were lysed, and equal protein amounts were used for Western blot analysis. Samples were probed for Nrf2 and NQO1 using actin as a loading control. The *lower panel* shows a densitometric analysis of Nrf2 (*left*) and NQO1 (*right*) protein normalized against β-actin. A representative experiment is shown.

To further support the impact of glucocorticoids on Nrf2 transcriptional activity and protein expression, we induced oxidative stress by incubating H4IIE cells with H_2_O_2_ and determined the suppressive effect of cortisol on Nrf2 and NQO1 protein levels. Cells were treated with vehicle (0.05% DMSO), cortisol (100 nM), sulforaphane (10 µM) or combinations of them, in the presence or absence of H_2_O_2_ (2 mM), followed by measurement of Nrf2 and NQO1 protein levels by Western blot analysis. H_2_O_2_ increased both basal and sulforaphane-induced Nrf2 and NQO1 protein. Cortisol treated cells showed reduced H_2_O_2_- and sulforaphane-inducible NQO1 and Nrf2 protein expression upon co-incubation with cortisol compared with cells treated with sulforaphane or H_2_O_2_ alone (Fig. 5).

### Downregulation of NQO1 protein levels in H4IIE cells expressing 11β-HSD1

**Figure 6:**
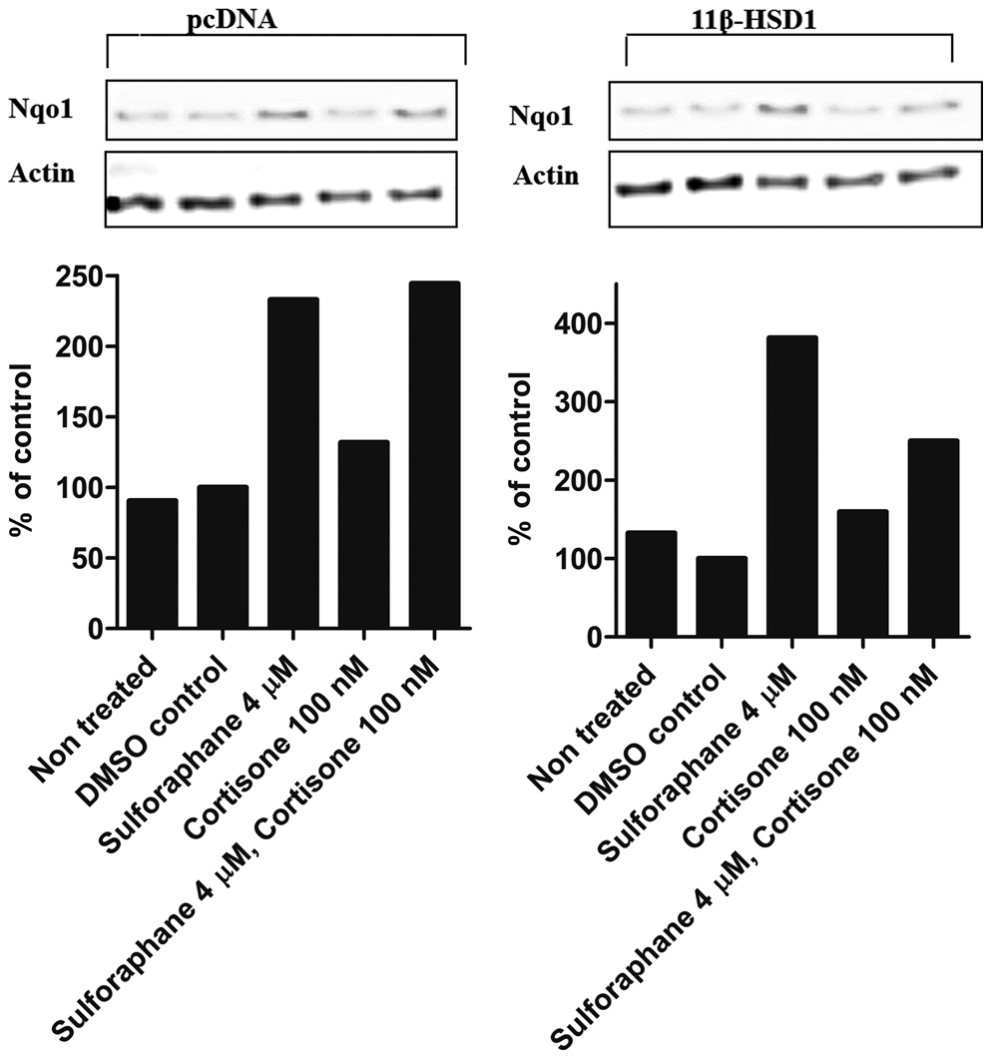
Suppression of NQO1 protein expression by cortisone in 11β-HSD1 expressing H4IIE cells but not in pCDNA3 transfected cells. H4IIE cells transiently transfected with either pCDNA3 or 11β-HSD1 were treated for 24 h with vehicle (DMSO), cortisone, sulforaphane or cortisone and sulforaphane (*upper panel*). Cells were lysed, and equal protein amounts were used for Western blot analysis. Samples were probed for NQO1 using actin as a loading control. *Lower panel*, densitometric analysis of NQO1 bands normalized against b-actin. Graphs are representative of three independent experiments.

To further support the suppressive effect of glucocorticoids on Nrf2 activity, we determined NQO1 protein levels in H4IIE cells transfected with either an empty vector (pcDNA3) or 11β-HSD1 expression plasmid. Cells were treated with vehicle (0.05% DMSO), cortisone (100 nM), sulforaphane (4 µM) or cortisone and sulforaphane, followed by measurement of NQO1 protein by Western blot analysis. In pcDNA3 transfected cells sulforaphane treatment enhanced NQO1 protein expression, whereas cortisone was ineffective (Fig. 6). Furthermore, cortisone alone did not affect basal protein expression. In contrast, 11β-HSD1 transfected cells showed reduced sulforaphane inducible NQO1 protein expression upon co-incubation with sulforaphane and 100 nM cortisone compared with cells treated with sulforaphane alone.

### Inhibition of 11β-HSD1 restores sulforaphane-induced NQO1 and GST2A mRNA expression in H4H1 cells

**Figure 7:**
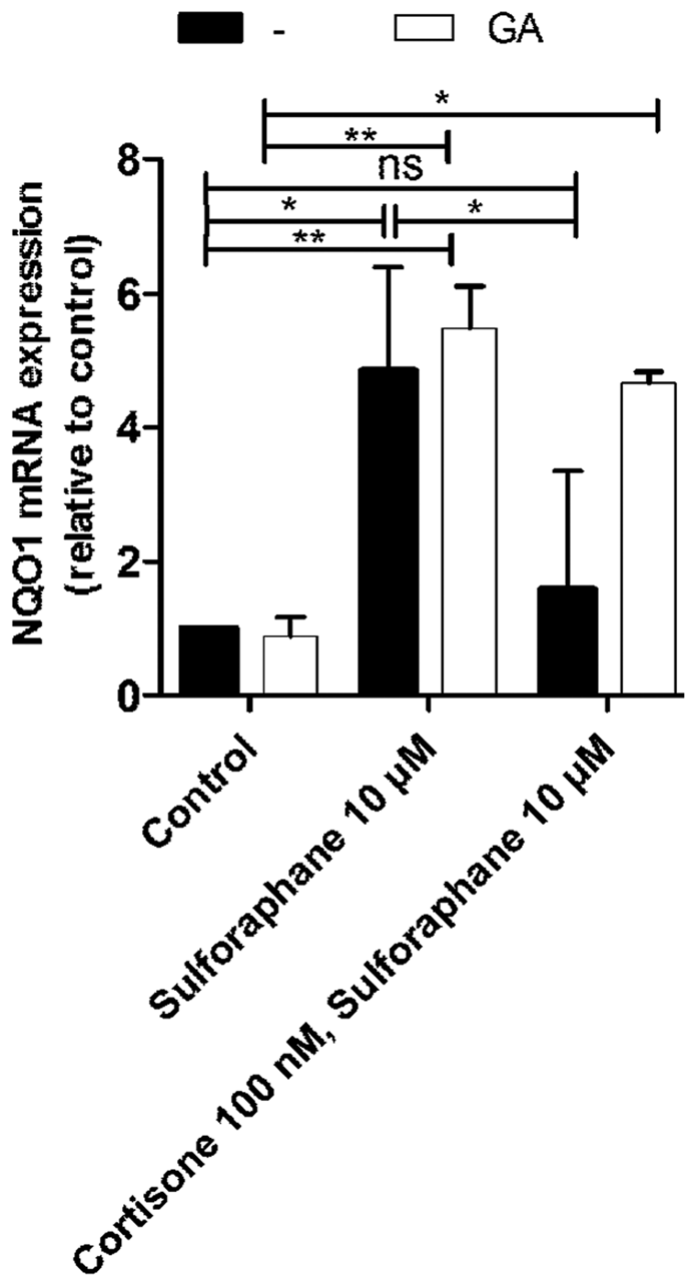
11β-HSD1-mediated suppression of Nrf2-induced NQO1 expression in H4H1 cells. H4H1 cells were incubated for 24 h at 37°C with 10 µM sulforaphane in the absence or presence of 100 nM cortisone and 1 µM glycyrrhetinic acid (GA), followed by quantification of mRNA levels by real-time RT-PCR. Data (mean ± S.D. from three independent experiments performed in triplicate) represent ratios of NQO1 mRNA to GAPDH control mRNA from treated cells normalized to the values obtained from cells incubated vehicle (DMSO). *, *p*<0.05, **, *p*<0.01, ***, *p*<0.001, *p*-values were obtained using one-way ANOVA followed by Bonferroni post-tests compared with vehicle control (DMSO).

Next, we investigated the impact of 11β-HSD1 activity on NQO1 mRNA expression in H4H1 cells (Fig. 7). Treatment with sulforaphane enhanced NQO1 mRNA expression, an effect which was significantly reduced upon simultaneous incubation of cells with sulforaphane and 100 nM cortisone. Similar observations were made for GST2A mRNA expression (Fig. S4). Treatment with cortisone suppressed basal expression of NQO1 and GST2A. In contrast, simultaneous incubation with cortisone and 1 µM of the 11β-HSD inhibitor glycyrrhetinic acid (GA) (Fig. 7), or the structurally unrelated 11β-HSD1 inhibitor T0504 (Fig. S4), showed no significant effect on basal or sulforaphane-induced NQO1 mRNA expression but completely reversed the suppressive effect by cortisone. Experiments with HPCT-1E3 cells with endogenous 11β-HSD1 confirmed the suppression of NQO1 and GST2A mRNA expression upon incubation with cortisone (Fig. S5).

### Impact of glucocorticoids on the susceptibility to H_2_O_2_

**Figure 8:**
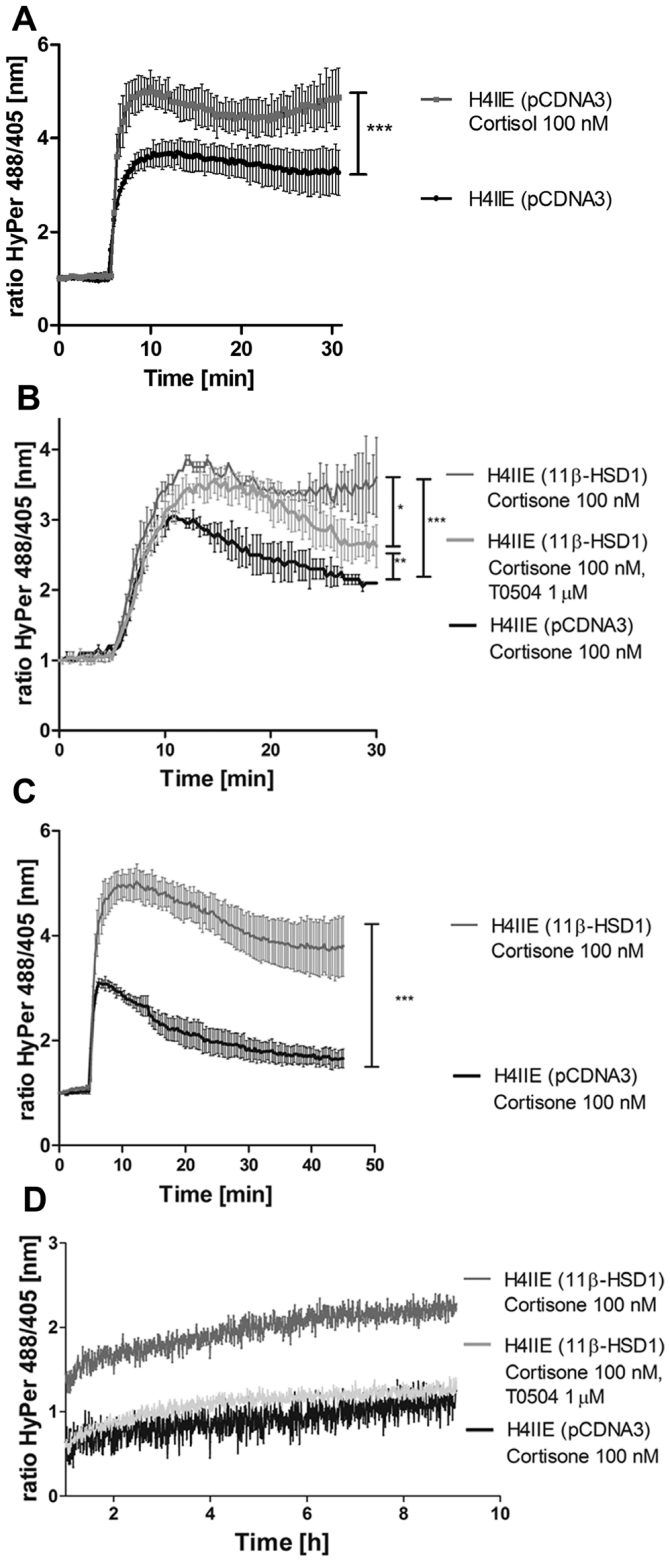
Increased susceptibility of 11β-HSD1 expressing cells to H_2_O_2_-induced oxidative stress. H4IIE cells transiently transfected with pCDNA3 (*A*), pCDNA3 or 11β-HSD1 (*B, C* and *D*) were treated for 24 h with vehicle, 100 nM cortisol (*A*), 100 nM cortisone (*C*) or simultaneously with cortisone and 1 µM T0504 (*B, D*). The medium was replaced by assay buffer (HBSS) containing 1 g/L glucose. Single cell real-time measurements were performed on a Leica SP5 confocal microscope. After 5 min baseline adaption, cells were exposed to a final concentration of 10 µM (*A, B*) or 100 µM (*C, D*) H**_2_**O**_2_** and responses were compared between differentially transfected or vehicle treated cells over a period of 30 min (*A, B*) or 45 min (*C*). Data represent mean ± SEM of seven different cells for each transfection. *, *p*<0.05, **, *p*<0.01, ***, p<0.001, ***p***-value was obtained using one-way ANOVA followed by Bonferroni post-tests compared with pcDNA3 transfected cells. To analyze the total cell population (*D*), H4IIE cells 4,000,000 cells/mL were resuspended in assay buffer. Suspensions of cells (100 µL) treated either with cortisone or cortisone and T0504 were transferred into a 96-well plate, centrifuged for 2 min at 180× g and challenged by adding 100 µL assay buffer containing 100 µM H**_2_**O**_2_**. Fluorescence was immediately measured after adding H_2_O_2_ and data were collected every 27 s at 37°C for 9 h. One of three representative experiments is shown (*D*).

To test the antioxidant response, we studied the sensitivity of H4IIE cells to H_2_O_2_. Accordingly, H4IIE cells were transiently transfected with a plasmid for the cytosolic H_2_O_2_-sensor HyPer and an empty expression vector (pCDNA3). Cells were treated with 100 nM cortisol for 24 h followed by treatment with H_2_O_2_ (100 µM) and real-time measurements of the HyPer response to the resulting cytosolic H_2_O_2_ concentration (Fig. 8A). H4IIE treated with cortisol showed an enhanced response to H_2_O_2_ compared with vehicle treated cells. Next, H4IIE cells were transiently transfected with plasmids for the cytosolic H_2_O_2_-sensor HyPer and either 11β-HSD1 or empty vector. Cells were treated with 100 nM cortisone for 24 h. The real-time measurements of the HyPer response to the cytosolic H_2_O_2_ concentration in empty vector control cells showed a rapid increase upon addition of 10 µM H_2_O_2_ (Fig. 8B). The initial peak was followed by a gradual decrease, reaching about 50% of the peak value after a recovery period of 25 min. In contrast, H4IIE transiently transfected with 11β-HSD1 showed an enhanced response to H_2_O_2_. Furthermore, 25 min after H_2_O_2_ injection, the HyPer fluorescence ratio was only marginally reduced, indicating an impaired ability to eliminate H_2_O_2_ in 11β-HSD1 expressing H4IIE cells. Simultaneous treatment with T0504 partially restored the cellular recovery from the H_2_O_2_ challenge (about 70% of the peak value after 25 min). Also, there was a tendency of lower peak response. Thus, the changes observed upon 11β-HSD1 expression were likely due to 11β-HSD1-mediated cortisol generation.

To more clearly visualize the observed differences upon 11β-HSD1 transfection, we performed a similar experiment using higher H_2_O_2_ concentration (Fig. 8C). H4IIE cells transfected with pCDNA3 or 11β-HSD1 were treated with cortisone, subjected to live microscopy, and challenged with 100 µM H_2_O_2_. As expected, the H_2_O_2_-induced increase in the HyPer fluorescence ratio was more rapid, and the inhibitory effect of 11β-HSD1 on the detoxification was more pronounced than in the experiment using 10 µM H_2_O_2_. Furthermore, while the signal reached almost baseline levels in control cells after 40 min of recovery, it was only slightly reduced in 11β-HSD1 expressing cells over the same time frame.

Next, we assessed the impact of 11β-HSD1 on the response of HyPer after a challenge with 100 µM H_2_O_2_ in a large cell population using fluorescence spectrophotometry (Fig. 8D). 11β-HSD1 expressing cells showed a more pronounced response to H_2_O_2_ and even after 9 h the HyPer signal did not return to baseline. Inhibition of 11β-HSD1 fully reversed this effect.

### Rat genome chip analysis

**Figure 9:**
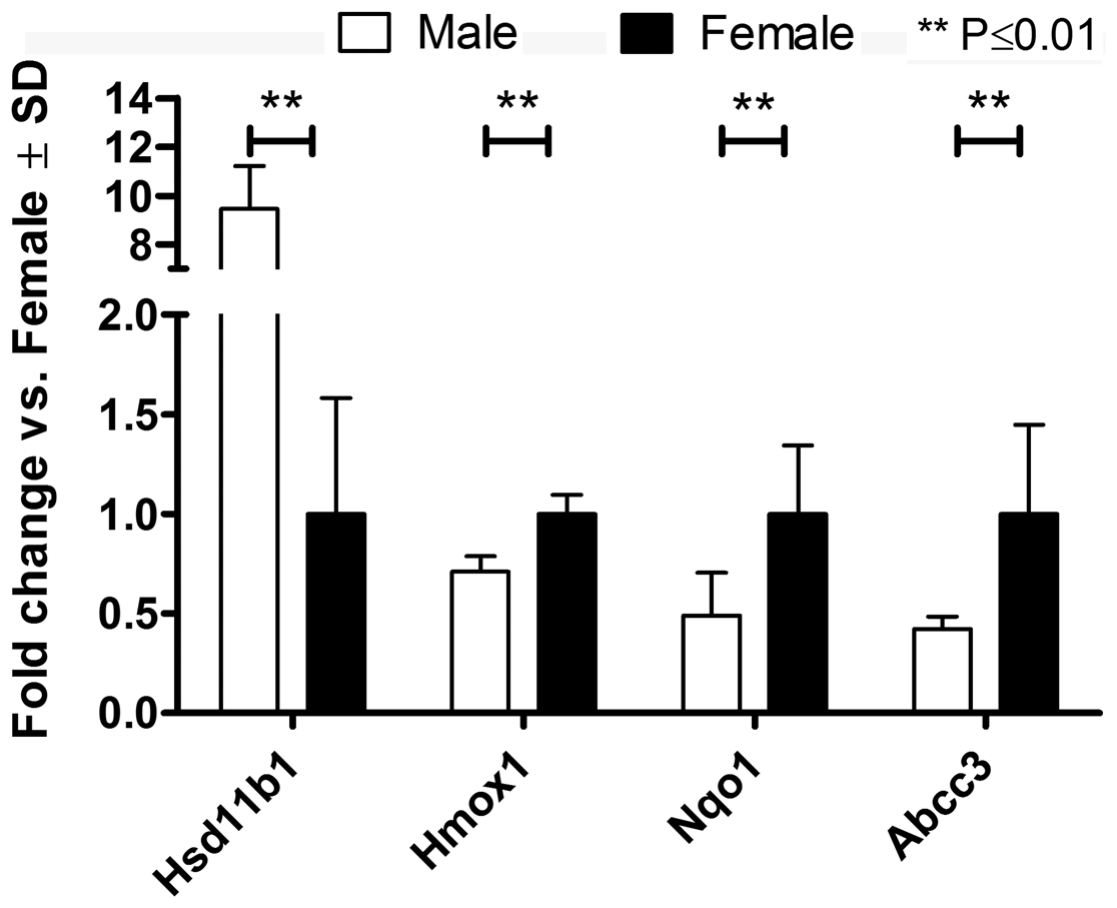
Rat Genome 230 2.0 Affymetrix chip analysis. RNA purified from whole liver tissues of ten male and ten female rats was hybridized to Rat Genome 230 2.0 Affymetrix chips. Gender-specific differences in the expression of the HSD11B1, HMOX1, NQO1 and ABCC3 genes were assessed. The data represent fold change in gene expression (male vs. female). The statistical relevance was assessed by multiple unpaired t-tests, with Benjamini Hochberg FDR multiple testing correction, *p*≤0.01.

Gender-specific differences in the expression of 11β-HSD1 and the Nrf2 target NQO1 have been reported [10], [19], [20]. Higher levels of 11β-HSD1 and lower levels of NQO1 have been observed in male compared with female rats. However, the expression analyses of 11β-HSD1 and NQO1 were performed independently and in different rat strains. Therefore, we examined whether there is an association between gender-specific 11β-HSD1 expression and Nrf2 target gene expression in the same strain of rat. RNA purified from whole liver tissues of ten male and ten female Han Wistar rats were hybridized to Rat Genome 230 2.0 Affymetrix chips (Table S1), and the analysis was restricted to a specific list of genes (Table S2). The full chip data analysis will be released elsewhere. In agreement with previous reports [19], [20], 11β-HSD1 expression was significantly higher (10.7-fold) in male compared with female rats (Fig. 9, for scatter plot see Fig. S6). Furthermore, we observed ten-fold higher 11β-HSD1 activity in whole liver homogenates of male compared with female rats. The elevated ability to generate active glucocorticoids correlated with a significantly lower (2.1-fold) NQO1 mRNA expression. Additionally, the Nrf2 target genes *HMOX1* (1.4-fold) and *ABCC3* (2.2-fold) were also significantly down regulated in male compared with female rats. Whereas the gender-specific differences in 11β-HSD1 expression levels correlated with differences in the basal expression of the Nrf2-dependent target genes, the mRNA levels of the genes encoding Nrf2 and GR did not differ between male and female rats.

## Discussion

To cope with the burden of reactive chemicals, sophisticated defense mechanisms emerged during evolution. Nrf2 plays a key role in regulating cellular responses to oxidative stress. Deficient or impaired Nrf2 function has been closely related with several major diseases such as rheumatoid arthritis [25], [26], diabetes [27], [28], Parkinson's [29] and various forms of cancer [30], [31]. Despite the key role of Nrf2 in redox regulation, its mechanism of action is highly complex and not fully understood [1], [17].

Another transcription factor, playing a key role in the regulation of many genes involved in biotransformation reactions and in the adaptation to altered energy demand, is the GR. Most of the reported studies addressing the impact of glucocorticoids on detoxification reactions used dexamethasone. However, dexamethasone is a highly potent synthetic glucocorticoid with clearly distinct properties compared with the endogenous glucocorticoid cortisol. Dexamethasone, an approximately ten-times more potent GR agonist than cortisol, activates also PXR at high concentrations [32]. PXR and its co-receptor retinoid-X receptor (RXR) are involved in the detoxification of xenobiotics by regulating the expression of phase I (CYP3A4) and phase II enzymes [32]. Some of the phase II enzymes regulated by Nrf2, including NQO1, contain both XRE and ARE motifs in their promoter regions [33]. PXR-mediated transcriptional regulation of GSTA2 by high concentrations of dexamethasone has been reported [34], [35]. Therefore, it is important to distinguish between effects of synthetic and endogenous glucocorticoids.

Since dexamethasone is not efficiently converted to 11-ketodexamethasone and the oxidized metabolite 11-ketodexamethasone still is a potent GR agonist [14], dexamethasone circumvents the important interconversion by 11β-HSD enzymes. Endogenous glucocorticoids can be metabolically inactivated by 11β-HSD2 in tissues such as kidney and colon and regenerated by 11β-HSD1 mainly in the liver. 11β-HSD1 activity depends on the availability of NADPH in the ER, which is determined by glucose-6-phosphate supply and the activity of the ER luminal enzyme H6PDH [36], [37], [38], [39].

A possible mechanism for the suppression of the Nrf2 pathway by the activated GR was proposed by Ki *et al.* [13]. GR activation by dexamethasone and its subsequent binding to glucocorticoid response elements (GRE) on the promoter of its target genes lead to the recruitment of transcription factors such as silencing mediator for retinoid and thyroid hormone receptors (SMRT) and nuclear receptor corepressor (NCoR). In this model, recruited SMRT acts as a corepressor by modulating chromatin structures by histone deacetylation. SMRT was further found to directly bind the Neh 4/5 domain of Nrf2, a protein motif essential for Nrf2 transactivation. The binding of SMRT to Neh 4/5 may then lead to the repression of Nrf2 target genes.

Here, we demonstrate that cortisol, and cortisone following conversion to cortisol by 11β-HSD1, suppress the Nrf2-dependent antioxidant cell defense pathway. The cortisone and cortisol concentrations used are in the physiological range, *i.e.* 100 nM. Under physiological conditions, glucocorticoid concentrations vary during circadian and ultradian rhythms, and it will be important to investigate in future studies the impact of glucocorticoid fluctuations on the Nrf2-dependent pathway *in vivo*. The effect of glucocorticoids on Nrf2 activity is expected to be highly tissue- and cell-specific, reflecting the expression of the respective 11β-HSD enzyme. Using H4IIE cells, we show that in the absence of 11β-HSD1, cortisol (with an IC_50_ of 10±5 nM) but not cortisone suppresses Nrf2 activity (Fig. 2, 4 and 6). In tissues expressing 11β-HSD2 (placenta, renal cortical collecting ducts, distal colon, several cancer cells) Nrf2 may be rather insensitive to glucocorticoids, which is in clear contrast to tissues expressing 11β-HSD1 (liver, adipose, hippocampal neurons, macrophages) that are able to generate active cortisol and are therefore exposed to higher concentrations of active glucocorticoids.

A recent study reported elevated hepatic 11β-HSD1 expression in patients with alcoholic liver disease (ALD) [40]. ALD-associated disorders include fatty liver, inflammation, and hepatocellular carcinoma in patients with liver cirrhosis [41]. Nrf2 prevents ethanol-induced liver injury by induction of the detoxification of acetaldehyde and inhibition of metabolite accumulation. Nrf2 knockout mice showed a dramatic increase in mortality following feeding with ethanol doses which were well tolerated in wild-type mice [42]. On a basic cellular level, our results suggest that pharmacological inhibition of 11β-HSD1 and antagonism of GR activity may be beneficial to restore the capacity of detoxification processes regulated by Nrf2. This was supported by transactivation assays, NQO1 mRNA expression levels (Fig. 2 and 7), and by the use of the intracellular redox-sensor HyPer reflecting the activity of Prx and HO-1 after H_2_O_2_ challenge (Fig. 8). In case of ALD, the authors claimed that 11β-HSD1 inhibition may represent a novel therapeutic approach to treat alcoholic pseudo-Cushing's [40]. We hypothesize that pharmacological inhibition of 11β-HSD1 may be beneficial for restoring hepatic detoxification capacity, at least in patients with ALD, which warrants clinical investigation.

In conclusion, the present study revealed a modulation of the Nrf2-dependent regulation of the antioxidant response pathway by glucocorticoids in hepatic H4IIE cells and suggests that elevated 11β-HSD1 activity may lead to impaired Nrf2-dependent cell defence. The physiological effects of endogenous glucocorticoids on the Nrf2-dependent detoxification may represent a novel fine-tuning mechanism by which glucocorticoids regulate the balance between energy supply, cell defense and ultimately cellular homeostasis. Inhibition of 11β-HSD1 and antagonism of GR restored the suppressive effect of elevated glucocorticoids on Nrf2-mediated target gene regulation. Future work should include animal experimentation and clinical studies to assess whether pharmacological inhibition of 11β-HSD1 or GR antagonism may improve Nrf2-dependent cell defense and whether such intervention may be beneficial for ALD patients or for patients with chronic inflammation such as diabetes or rheumatoid arthritis.

## Materials and Methods

### Materials

[1,2-^3^H]-cortisone was purchased from American Radiolabeled Chemicals (St. Louis, MO), cell culture media from Invitrogen (Carlsbad, CA), sulforaphane from Sigma-Aldrich (Buchs, Swizerland) and all other chemicals from Fluka AG (Buchs, Switzerland). The luciferase reporter plasmid containing an eight times repeated antioxidant response element (ARE8L-reporter), human Nrf2, human recombinant GR-α and human 11β-HSD1 expression constructs have been described earlier [21], [43]. Human HEK-293 cells (No CRL-1573) and rat H4IIE hepatoma cells (No CRL-1600) were obtained from ATCC through LGC Standards S.a.r.l., Molsheim Cedex, France. H4IIE cells stably expressing murine 11β-HSD1 (clone H4H1) were described earlier [24]. The work was performed under the approval number A070126 from the Eidgenössisches Departement für Umwelt, Verkehr, Energie und Kommunikation UVEK, Bundesamt für Umwelt BAFU, Switzerland.

### Cell culture and transfection

HEK-293 ells (100'000 cells/well) were cultured in Dulbecco's modified Eagle medium (DMEM) supplemented with 10% fetal bovine serum, 4.5 g/L glucose, 50 U/mL penicillin/streptomycin, 2 mM glutamine, and 1 mM HEPES, pH 7.4, were seeded in poly-L-lysine coated 24-well plates and incubated for 16 h. Cells were transfected using calcium phosphate precipitation with ARE8L-reporter (0.20 µg/well), pCMV-LacZ galactosidase transfection control (0.03 µg/well), GR (0.20 µg/well), Nrf2 (0.20 µg/well), and either 11β-HSD1 (0.20 µg/well) or empty pcDNA3 vector (0.20 µg/well).

H4IIE and H4H1 cells were cultured in antibiotic-free DMEM supplemented as given above. H4IIE and H4H1 cells were transfected using electroporation (Neon™, Invitrogen) according to the manufacturer. Cells were trypsinized, washed once with PBS, centrifuged for 2 min at 100×g and resuspended in 288 µL resuspension buffer with the final transfection density of 1×10^6^ cells/mL. Cells were subjected to a single pulse using a 100 µL gold tip at 1375 V for 30 ms, with a total amount of 2.5 µg DNA consisting of ARE8L-reporter (2 µg) and pCMV-LacZ galactosidase transfection control (0.5 µg). To assess the impact of 11β-HSD1, H4IIE cells were also transiently transfected with plasmids for 11β-HSD1 (2 µg) or pcDNA3 control (2 µg), ARE8L-reporter (2 µg) and pCMV-LacZ (0.5 µg).

To assess the susceptibility of H4IIE cells to H_2_O_2_-mediated redox sensitivity, cells were transfected with either pcDNA3 (2 µg) or 11β-HSD1 (2 µg) and 4 µg of the cytosolic HyPer-plasmid [44]. Cells (100'000 cells/well) were cultured in DMEM for 24 h at 37°C in six-well plates containing glass coverslips. Cells were washed once with charcoal-treated, steroid-free DMEM (DMEMct) and incubated for another 3 h. The culture medium was replaced with fresh DMEMct containing cortisone (100 nM) with or without 11β-HSD1 inhibitor T0504 (1 µM) [45] and cells were cultured for another 24 h. To evaluate whether GR transactivation is influenced by H_2_O_2_-mediated oxidative stress, H4IIE cells were transfected with TAT3-TATA (2 µg) and pCMV-LacZ transfection control (0.5 µg).

To further elaborate the role of basal 11β-HSD1 expression, HPCT-1E3 cells endogenously expressing 11β-HSD1, were cultured in DMEM supplemented with 10% fetal bovine serum, 4.5 g/L glucose, 50 U/mL penicillin/streptomycin, 1.5 µM dexamethasone, 10 mg/mL insulin and inosine and 4 mM glutamine (Dex-DMEM) [46].

### Construction of H4IIE cells stably expressing the ARE8L-reporter

H4IIE cells at passage four were co-transfected by electroporation with the ARE8L-reporter plasmid and pcDNA3.1(+) containing a neomycin resistance gene. Transfected cells were screened upon administration of 2 mg/mL G418 (Invitrogen, Carlsbad, CA) over two weeks. Single clones stably expressing ARE8L were selected from neomycin resistant H4IIE cells. Isolated single clones were verified for their luciferase activity in transactivation assays after stimulation with sulforaphane (10 µM) for 24 h at 37°C. Cell clones expressing high levels of ARE8L were further sub-cultivated for two weeks with DMEM containing 1 mg/mL G418 to maintain selective pressure. The single clone designated as ARECS3 was used for further experiments.

### Nrf2 and GR transactivation assays

Cells were washed twice with DMEM 6 h post-transfection, followed by incubation for 24 h at 37°C in antibiotic-free DMEM to allow sufficient expression. Cells were washed once with steroid- and serum-free DMEM (DMEMsf) and incubated for 3 h at 37°C. For Nrf2 transactivation the culture medium was replaced with fresh DMEMsf containing sulforaphane (10 µM), T0504 (1 µM), RU-486 (1 µM) and combinations of them, in the presence or absence of glucocorticoids (100 nM). For IC_50_ determination, H4IIE cells were simultaneously treated with sulforaphane (10 µM) and increasing concentrations of cortisol (4–972 nM). The influence of oxidative stress on GR transactivation was assessed in H4IIE cells treated with cortisol (100 nM), sulforaphane (10 µM) and combinations of them, in the presence or absence of H_2_O_2_ (2 mM). Activation of Nrf2 by H_2_O_2_-mediated oxidative stress was assessed in ARECS3 cells stably expressing the ARE8L reporter construct. Cells were treated with vehicle or sulforaphane (10 µM) in the presence or absence of H_2_O_2_ (2 mM). After incubation for another 24 h, cells were washed once with PBS, lysed with 60 µL lysis buffer of the Tropix kit (Applied Biosystems, Foster City, CA) supplemented with 0.5 mM dithiothreitol, and frozen. Lysates were analyzed for luciferase activity using a home-made luciferine-solution [14]. β-galactosidase activity was analyzed using the Tropix kit according to the manufacturer.

### Analysis of mRNA expression by real-time RT-PCR

H4IIE and H4H1 cells (500'000 cells/well) were cultured in 24-well plates with DMEM for 12 h at 37°C. Cells were washed once with DMEMsf and incubated for another 3 h at 37°C. The culture medium was replaced with fresh DMEMsf containing sulforaphane (10 µM), T0504 (1 µM), RU-486 (1 µM) or combinations of them, in the presence or absence of glucocorticoids (100 nM), followed by incubation for another 24 h at 37°C. The role of endogenous 11β-HSD1 expression was assessed in HPCT-1E3 cells. HPCT-1E3 cells (1'000'000 cells/well) seeded in six-well plates were cultivated for 12 h at 37°C in DMEM supplemented with 1.5 µM dexamethasone. Cells were washed twice with DMEMct and incubated for 3 h at 37°C. The culture medium was replaced with fresh DMEMct and cells were cultivated for another 24 h at 37°C. The influence of oxidative stress on 11β-HSD1 expression was assessed in HPCT-1E3 cells treated with H_2_O_2_ (2 mM) for 24 h at 37°C. Total mRNA was extracted using the Trizol method (Invitrogen, Carlsbad, CA). Total mRNA (2 µg) was reverse transcribed to cDNA using the Superscript-III First-Strand Synthesis System and oligo-dT (Invitrogen). Relative quantification of mRNA expression levels was performed by RT-PCR on a RotorGene 6000 (Corbett, Australia) using the KAPA SYBR® FAST qPCR Kit (Kapasystems, Boston, MA). Relative gene expression compared with the internal control GAPDH was determined using the delta-delta-CT method.

### Western blot analysis

H4IIE cell lysates (10 µg of protein) were obtained upon lysis in RIPA buffer (25 mM Tris-HCl, pH 7.6, 150 mM NaCl, 1% NP-40, 1% sodium deoxycholate, 0.1% SDS). Samples were resolved by electrophoresis on a 12% polyacrylamide gel. Proteins were transferred to nitrocellulose membranes, which were blocked overnight at 4°C using 10% fat-free milk in TBS containing 0.1% Tween-20. For NQO1 protein determination membranes were probed for 1 h at 4°C with anti-NQO1 (1∶2000; Abcam, ab2346) and anti-actin (1∶2000; santa-cruz, sc-1616) primary antibodies in TBS containing 0.1% Tween-20 and 2% fat-free milk. For Nrf2 protein determination membranes were probed for 2 h at 4°C with anti-Nrf2 rabbit polyclonal (1∶2000; Abcam, ab53019) and anti-actin (1∶2000; santa-cruz, sc-1616) primary antibodies in TBS containing 0.1% Tween-20 and 5% fat-free milk. Membranes were washed 4×15 min with TBS, 0.1% Tween-20 at 25°C and then probed for 1 h at 4°C with anti-goat (1∶2000, DAKO, p0449) and anti-rabbit (1∶2000, Sigma, A0545) HRP-linked secondary antibodies in TBS containing 0.1% Tween-20 and 2% fat-free milk. Membranes were washed 4×15 min with TBS, 0.1% Tween-20 and proteins were visualized by Enhanced Chemiluminescence plus (GE Healthcare) using a Fujifilm LAS-4000 detection system (Bucher Biotec, Basel, Switzerland).

### Detection of H_2_O_2_ sensitivity by fluorescence microscopy

A Leica SP5 confocal microscope was used for single cell imaging. Scanning was performed at 400 Hz in a 512×512 pixel format. Excitation of the protonated and charged form of HyPer [44] were performed using the 405 nm and the 488 nm laser lines, respectively. Emission was recorded between 500–554 nm. Pictures were taken every 20 s and 488/405 emission intensity ratios calculated. For analyzing the total cell population, the Gemini-EM spectrofluorometer (Molecular Devices, Sunnyvale, CA) was used. Excitation of the protonated form of HyPer was measured at 420 nm. Excitation of the charged form was detected at 490 nm, and emission was recorded at 535 nm. Data were recorded every 27 s over 9 h at 37°C.

### Rat genome chip analysis

RNA purified from whole liver tissues of 20 Han Wistar rats (animals listed in Table S1) were hybridized to Rat Genome 230 2.0 chips (Affymetrix, Santa Clara, CA) for characterization of gene expression. Hybridization mixtures were prepared using the 3′-IVT Express Kit (Affymetrix) to accommodate 10 µg of labeled cDNA in 200 µL of hybridization mix. Rat Genome 230 2.0 Arrays were hybridized, revealed and washed according to the Affymetrix protocol. GeneChips were scanned using the Affymetrix 3000 scanner and images were converted to files (*.cel). The raw data were analyzed by GeneSpring GX 11.5.1 (Agilent) using the software default settings for Affymetrix expression chips. Raw data were pre-processed by Robust Multi-array Analysis (RMA) algorithm (including Quantile normalization), Log transformed (Table S2) and then pre-filtered within the 20–100 percentiles assuming the median as baseline (raw data). Of the 31099 probe-sets in Rat Genome 230 2.0 chip, 27359 have passed the pre-filter. Correlation plot (not shown) shows the correlation analysis (heat map, Pearson correlation factor) across arrays: high correlation degree is indicative of good experimental execution and high reproducibility. The internal quality controls of Affymetrix chips showed no abnormalities in the hybridization processes (not shown). Both analyses were part of the default quality controls [47], [48], [49], [50], [51].

## Supporting Information

Table S1 Han Wistar rats (disease status normal) used for RNA purification of whole liver tissues (Rat Genome 230 2.0 Affymetrix chip analysis).
(DOC)

Table S2 Raw and normalized gene expression data from Affymetrix chips.
(DOC)

Figure S1 **Increase of Nrf2 and NQO1 protein expression by proteasome inhibitor MG132 in H4IIE cells.** To see whether proteasome inhibition would increase Nrf2 protein expression, as well as that of the transcriptionally regulated target NQO1, we incubated H4IIE cells for 4 h with vehicle (0.05% DMSO) or 10 µM of the proteasome inhibitor MG132 (*upper panel*). Cells were lysed, and equal protein amounts were used for Western blot analysis. Samples were probed for Nrf2 and NQO1, using actin as a loading control. *Lower panel*, densitometric analysis of Nrf2 (*left*) and NQO1 (*right*) protein normalized against β-actin. MG132 increased Nrf2 and NQO1 protein approximately two-fold. Graphs are representative of two independent experiments.
(TIF)

Figure S2 **Sulforaphane does not inhibit 11β-HSD1 activity.** Enzymatic activities were determined in intact H4H1 cells stably expressing 11β-HSD1. Briefly, 30,000 cells/well were seeded in 96-well plates (Becton-Dickinson, Basel, Switzerland). Cells were washed once 24 h later with 50 µL DMEMsf and incubated for another 3 h at 37°C. The medium was replaced by 40 µL fresh medium containing either vehicle or sulforaphane, and 10 µL medium containing 10 nCi [1,2-^3^H]-cortisone and 50 nM unlabeled cortisone to assess 11β-HSD1 reductase activity. Cells were incubated for 40 min at 37°C and reactions stopped by adding 2 mM of unlabeled cortisone and cortisol in methanol, followed by separation of steroids by thin layer chromatography and determination of the conversion of radiolabeled substrate by scintillation counting. 11β-HSD1 activity after sulforaphane treatment was indistinguishable from that of control cells treated with vehicle (DMSO). Data represent mean ± SD from at least three independent experiments measured in triplicate. *P*-value was determined using unpaired, two-tailed student t-test (*ns*, not significant).
(TIF)

Figure S3 ***HSD11B1*** **mRNA expression and activity is unaffected by H_2_O_2_.** To assess whether *HSD11B1* mRNA expression is influenced by H_2_O_2_, HPCT-1E3 cells were cultivated for 24 h at 37°C in the presence of vehicle (0.05% DMSO) or H_2_O_2_ (2 mM) (*A*). Total mRNA was extracted using the Trizol method (Invitrogen, Carlsbad, CA). Total mRNA (2 µg) was reverse transcribed to cDNA using the Superscript-III First-Strand Synthesis System and oligo-dT (Invitrogen). Relative quantification of *HSD11B1* mRNA expression was performed by RT-PCR on a RotorGene 6000 (Corbett, Australia) using the KAPA SYBR® FAST qPCR Kit (Kapasystems, Boston, MA). Relative *HSD11B1* gene expression compared with the internal control GAPDH was determined using the delta-delta-CT method. Data (mean ± SD from two independent experiments measured in triplicate) represent ratios of 11ß-HSD1 mRNA to GAPDH control mRNA from treated cells normalized to the values obtained from cells incubated with vehicle (DMSO). Enzymatic activities were determined in intact HPTC-1E3 cells expressing endogenous 11β-HSD1 that were treated with vehicle (DMSO), 2 mM H_2_O_2_ (*B*) or 1 µM RU-486 (*C*). Briefly, 50'000 cells/well were seeded in 96-well plates (Becton-Dickinson, Basel, Switzerland). Cells were washed twice 12 h later with 50 µL DMEMct and incubated for another 24 h at 37°C. The medium was replaced by 40 µL fresh medium containing either vehicle, H_2_O_2_ (2 mM) or RU-486 (1 µM), and 10 µL medium containing 10 nCi [1,2-^3^H]-cortisone and 50 nM unlabeled cortisone to assess 11β-HSD1 reductase activity. Cells were incubated for 4 h at 37°C and reactions stopped by adding 2 mM of unlabeled cortisone and cortisol in methanol, followed by separation of steroids by thin layer chromatography and determination of the conversion of radiolabeled substrate by scintillation counting. Oxidative stress induced by H_2_O_2_ neither changed the expression of 11β-HSD1 mRNA nor its activity in HPCT-1E3 cells. Furthermore, the GR inhibitor RU-486 did not affect 11β-HSD1 activity. Data represent mean ± SD from three independent experiments.
(TIF)

Figure S4 **The 11β-HSD1 inhibitor T0504 restores NQO1 mRNA and GST2A mRNA expression in H4H1 cells.** H4H1 cells (500'000 cells/well) were cultured in 24-well plates with DMEM for 12 h at 37°C. Cells were washed once with DMEMsf and incubated for another 3 h at 37°C. The culture medium was replaced with fresh DMEMsf containing sulforaphane (10 µM), T0504 (1 µM) or combinations of them, in the presence or absence of cortisone (100 nM), followed by incubation for 24 h at 37°C. Total mRNA was extracted using the Trizol method (Invitrogen, Carlsbad, CA). Total mRNA (2 µg) was reverse transcribed to cDNA using the Superscript-III First-Strand Synthesis System and oligo-dT (Invitrogen). Relative quantification of mRNA expression levels was performed by RT-PCR on a RotorGene 6000 (Corbett, Australia) using the KAPA SYBR® FAST qPCR Kit (Kapasystems, Boston, MA). Relative gene expression compared with the internal control GAPDH was determined using the delta-delta-CT method. Incubation with cortisone (100 nM) suppressed basal (0.5-fold and 0.3-fold) as well as sulforaphane-induced (1.5-fold and 2-fold) NQO1 (*A*) and GST2A mRNA (*B*) expression in H4H1 cells. Simultaneous treatment with the 11β-HSD1 inhibitor T0504 restored NQO1 and GST2A mRNA expression and fold activation was almost indistinguishable from cells treated with sulforaphane alone (2-fold and 2.8-fold) or vehicle control expression levels. Data represent mean ± SD from a representative experiment measured in triplicate.
(TIF)

Figure S5 **Incubation with cortisone suppresses NQO1 and GST2A mRNA expression in HPCT-1E3 cells with endogenous 11β-HSD1 activity.** HPCT-1E3 cells (1'000'000 cells/well) were cultured in 6-well plates with dexamethasone supplemented DMEM for 12 h at 37°C. Cells were washed twice with DMEMct and incubated for another 3 h at 37°C. The culture medium was replaced with fresh DMEMct and cells were cultivated for another 24 h at 37°C. To assess whether basal 11β-HSD1 activity suppresses Nrf2, the culture medium was replaced with fresh DMEMct containing vehicle, sulforaphane (10 µM), T0504 (1 µM), and combinations of them, in the presence or absence of cortisone (100 nM) and cells were incubated for another 24 h at 37°C. Total mRNA was extracted using the Trizol method (Invitrogen, Carlsbad, CA). Total mRNA (2 µg) was reverse transcribed to cDNA using the Superscript-III First-Strand Synthesis System and oligo-dT (Invitrogen). Relative quantification of Nrf2 mRNA expression was performed by RT-PCR on a RotorGene 6000 (Corbett, Australia) using the KAPA SYBR® FAST qPCR Kit (Kapasystems, Boston, MA). Relative NQO1 mRNA and GST2A mRNA gene expression compared with the internal control GAPDH was determined using the delta-delta-CT method. NQO1 (*A*) and GST2A (*B*) mRNA expression levels were induced by sulforaphane (10 µM) 4-fold and 14-fold, respectively, in HPCT-1E3 cells. Cortisone (100 nM) treatment reduced both NQO1 and GST2A mRNA expression levels. Data represent mean ± SD from a representative (one out of three) experiment performed in triplicate.
(TIF)

Figure S6 **Expression of the Nrf2-dependent genes NQO1, Abcc3 and Hmox1 in comparison to** ***HSD11B1*** **relative expression.** *HSD11B1* relative gene expression was evaluated in comparison to NQO1, Abcc3 and Hmox1, in each animal, by scatter plot. RNA purified from whole liver tissues of 20 Han Wistar rats (animals listed in Table S1) were hybridized to Rat Genome 230 2.0 chips (Affymetrix, Santa Clara, CA) for characterization of gene expression. Hybridization mixtures were prepared using the 3′-IVT Express Kit (Affymetrix) to accommodate 10 µg of labeled cDNA in 200 µL of hybridization mix. Rat Genome 230 2.0 Arrays were hybridized, revealed and washed according to the Affymetrix protocol. GeneChips were scanned using the Affymetrix 3000 scanner and images were converted to files (*.cel). The raw data were analyzed by GeneSpring GX 11.5.1 (Agilent) using the software default settings for Affymetrix expression chips (Table S2). Raw data were pre-processed by Robust Multi-array Analysis (RMA) algorithm (including Quantile normalization), Log transformed and then pre-filtered within the 20–100 percentile assuming the median as baseline (raw data). Of the 31099 probe-sets in Rat Genome 230 2.0 chip, 27359 have passed the pre-filter. Correlation plot (not shown) shows the correlation analysis (heat map, Pearson correlation factor) across arrays: high correlation degree is indicative of good experimental execution and high reproducibility. The internal quality controls of Affymetrix chips showed no abnormalities in the hybridization processes (not shown). Both analyses were part of the default quality controls. Linear regression analysis was used to obtain the overall correlation (R2) of *HSD11B1* expression with other genes (*HSD11B1* vs. Nqo1 R2 = 0.546, p value = 2.00E-004; *HSD11B1* vs. Abcc3 R2 = 0.407, p value = 2.47E-003; *HSD11B1* vs. Hmox1 R2 = 0.692, p value = 5.38E-006). The statistical relevance was assessed by multiple unpaired *t*-tests, with Benjamini Hochberg FDR multiple testing correction, *p*≤0.05.
(TIF)
