# Supplementary material for: Suppression of the Nrf2-Dependent Antioxidant Response by Glucocorticoids and 11β-HSD1-Mediated Glucocorticoid Activation in Hepatic Cells
Source: PLoS One. 2012 May 11;7(5):e36774. doi: 10.1371/journal.pone.0036774 (PMC3350474; doi:10.1371/journal.pone.0036774)
Supplement: Table S1 — Han Wistar rats (disease status normal) used for RNA purification of whole liver tissues (Rat Genome 230 2.0 Affymetrix chip analysis). (DOC) [file pone.0036774.s001.doc]

**Table S1: Han Wistar rats (disease status normal) used for RNA purification of whole liver tissues (Rat Genome 230 2.0 Affymetrix chip analysis)**

| **Array experiment NUID** | **Organ** | **Donor species** | **Gender** |
| --- | --- | --- | --- |
| **NUID-0000-0100-9757.cel** | **Liver** | **Rattus norvegicus** | **male** |
| **NUID-0000-0100-9768.cel** | **Liver** | **Rattus norvegicus** | **male** |
| **NUID-0000-0100-9779.cel** | **Liver** | **Rattus norvegicus** | **male** |
| **NUID-0000-0100-9790.cel** | **Liver** | **Rattus norvegicus** | **male** |
| **NUID-0000-0100-9801.cel** | **Liver** | **Rattus norvegicus** | **male** |
| **NUID-0000-0100-9812.cel** | **Liver** | **Rattus norvegicus** | **male** |
| **NUID-0000-0100-9823.cel** | **Liver** | **Rattus norvegicus** | **male** |
| **NUID-0000-0100-9834.cel** | **Liver** | **Rattus norvegicus** | **male** |
| **NUID-0000-0100-9836.cel** | **Liver** | **Rattus norvegicus** | **male** |
| **NUID-0000-0100-9758.cel** | **Liver** | **Rattus norvegicus** | **male** |
| **NUID-0000-0100-9759.cel** | **Liver** | **Rattus norvegicus** | **female** |
| **NUID-0000-0100-9760.cel** | **Liver** | **Rattus norvegicus** | **female** |
| **NUID-0000-0100-9761.cel** | **Liver** | **Rattus norvegicus** | **female** |
| **NUID-0000-0100-9762.cel** | **Liver** | **Rattus norvegicus** | **female** |
| **NUID-0000-0100-9763.cel** | **Liver** | **Rattus norvegicus** | **female** |
| **NUID-0000-0100-9764.cel** | **Liver** | **Rattus norvegicus** | **female** |
| **NUID-0000-0100-9765.cel** | **Liver** | **Rattus norvegicus** | **female** |
| **NUID-0000-0100-9766.cel** | **Liver** | **Rattus norvegicus** | **female** |
| **NUID-0000-0100-9767.cel** | **Liver** | **Rattus norvegicus** | **female** |
| **NUID-0000-0100-9769.cel** | **Liver** | **Rattus norvegicus** | **female** |
