# Supplementary material for: Suppression of the Nrf2-Dependent Antioxidant Response by Glucocorticoids and 11β-HSD1-Mediated Glucocorticoid Activation in Hepatic Cells
Source: PLoS One. 2012 May 11;7(5):e36774. doi: 10.1371/journal.pone.0036774 (PMC3350474; doi:10.1371/journal.pone.0036774)
Supplement: Table S2 — Raw and normalized gene expression data from Affymetrix chips. (DOC) [file pone.0036774.s002.doc]

**Table S2.** Raw and normalized gene expression data from Affymetrix chips.

|  | **Hsd11b1 - 1386956_at** | | **Hmox1 - 1370080_at** | | **Nqo1 - 1387599_a_at** | | **Abcc3 - 1369698_at** | |
| --- | --- | --- | --- | --- | --- | --- | --- | --- |
| **Sample** | **Normalized (Log scale)** | **Raw** | **Normalized** | **Raw** | **Normalized** | **Raw** | **Normalized** | **Raw** |
| **NUID-0000-0100-9759.cel** | -2.5507774 | 344.5947 | 0.007117271 | 167.36446 | 1.0312157 | 824.8311 | 1.8649292 | 91.81916 |
| **NUID-0000-0100-9760.cel** | -2.880866 | 274.1208 | 0.40091228 | 219.89095 | 0.37915802 | 524.8994 | 0.13122511 | 27.60809 |
| **NUID-0000-0100-9761.cel** | -2.204317 | 438.1306 | 0.18014908 | 188.69092 | 1.3176947 | 1006.014 | 0.4270625 | 33.89162 |
| **NUID-0000-0100-9762.cel** | -2.1748857 | 447.1603 | 0.3478694 | 211.95314 | 0.46074486 | 555.4388 | 0.78475285 | 43.4278 |
| **NUID-0000-0100-9763.cel** | -2.0782614 | 478.1345 | 0.18465519 | 189.2812 | 0.73599625 | 672.1948 | 0.6600952 | 39.83292 |
| **NUID-0000-0100-9764.cel** | -0.77149105 | 1182.843 | 0.32789516 | 209.03885 | 0.77137184 | 688.881 | 0.34013748 | 31.90989 |
| **NUID-0000-0100-9765.cel** | -2.8975754 | 270.9643 | 0.24743891 | 197.70033 | -0.3076744 | 326.0761 | 0.64336395 | 39.37364 |
| **NUID-0000-0100-9766.cel** | -1.8655233 | 554.1032 | 0.28567648 | 203.01016 | 0.8145685 | 709.8192 | 0.7603049 | 42.69807 |
| **NUID-0000-0100-9767.cel** | -1.3342857 | 800.7724 | -0.00711679 | 165.72136 | 0.016942024 | 408.3554 | 0.56756306 | 37.35831 |
| **NUID-0000-0100-9769.cel** | -0.7321205 | 1215.567 | 0.35834265 | 213.49734 | -0.01694202 | 398.8762 | 1.5986471 | 76.34394 |
| **NUID-0000-0100-9757.cel** | 1.6832075 | 6484.325 | -0.40771866 | 125.54092 | -1.4761958 | 145.0638 | -0.62458897 | 16.34985 |
| **NUID-0000-0100-9758.cel** | 1.1914473 | 4611.376 | -0.18015862 | 146.98993 | 0.18157291 | 457.717 | -0.36366463 | 19.59114 |
| **NUID-0000-0100-9768.cel** | 1.4520512 | 5524.339 | -0.29570675 | 135.6763 | -0.5823755 | 269.5415 | -0.31445122 | 20.27097 |
| **NUID-0000-0100-9779.cel** | 0.73212147 | 3353.971 | -0.23160219 | 141.84091 | 0.30801678 | 499.6438 | -0.86184144 | 13.87055 |
| **NUID-0000-0100-9790.cel** | 1.5726709 | 6006.068 | -0.18002605 | 147.00343 | -0.09931564 | 376.7395 | -0.4730301 | 18.1609 |
| **NUID-0000-0100-9801.cel** | 1.5397806 | 5870.691 | -0.3363614 | 131.90636 | -1.6561723 | 128.0503 | -0.23445559 | 21.42671 |
| **NUID-0000-0100-9812.cel** | 1.8032742 | 7047.077 | -0.35024548 | 130.64305 | -1.2750635 | 166.7654 | -0.26452208 | 20.98479 |
| **NUID-0000-0100-9823.cel** | 1.5393696 | 5869.019 | -0.40370178 | 125.89092 | -0.47269154 | 290.8331 | -0.15558243 | 22.63074 |
| **NUID-0000-0100-9834.cel** | 1.6913786 | 6521.159 | 0.08298111 | 176.40079 | -0.04141522 | 392.1669 | -0.13122511 | 23.01606 |
| **NUID-0000-0100-9836.cel** | 1.4545965 | 5534.095 | -0.30933905 | 134.40034 | -0.55788136 | 274.1569 | -0.29682255 | 20.52018 |
