# Supplementary figures and images for: Suppression of the Nrf2-Dependent Antioxidant Response by Glucocorticoids and 11β-HSD1-Mediated Glucocorticoid Activation in Hepatic Cells
Source: PLoS One. 2012 May 11;7(5):e36774. doi: 10.1371/journal.pone.0036774 (PMC3350474; doi:10.1371/journal.pone.0036774)

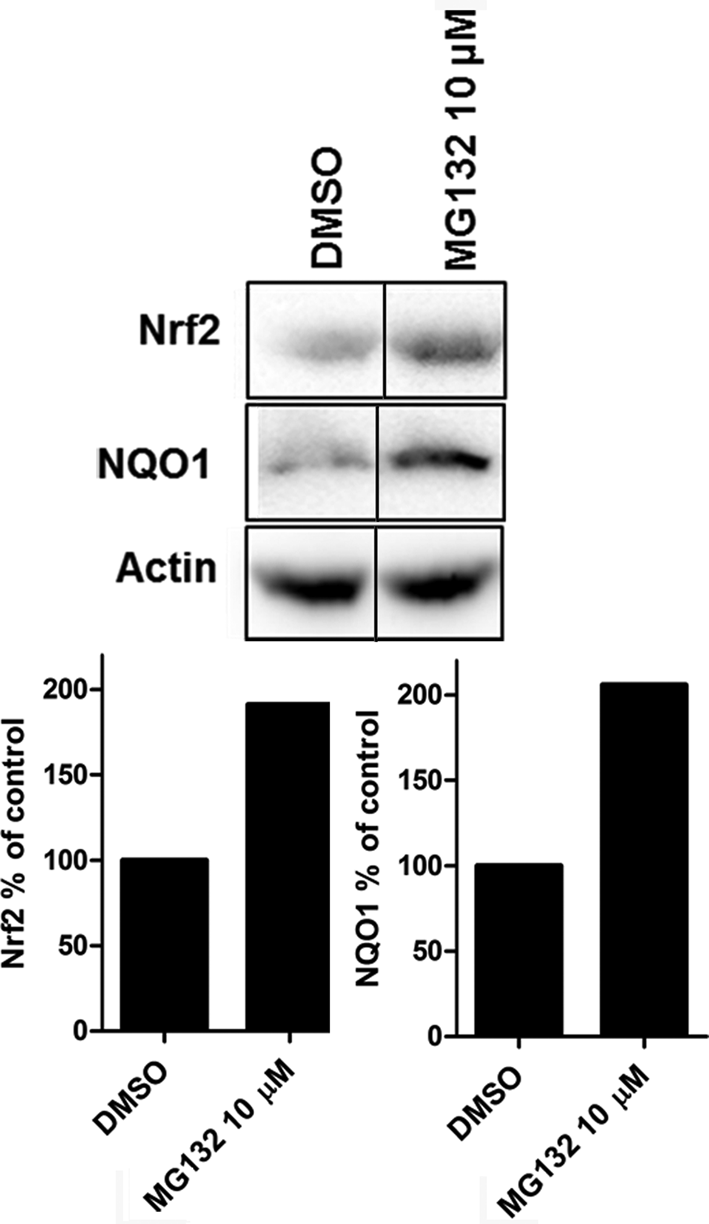

Supplement: Figure S1 — Increase of Nrf2 and NQO1 protein expression by proteasome inhibitor MG132 in H4IIE cells. To see whether proteasome inhibition would increase Nrf2 protein expression, as well as that of the transcriptionally regulated target NQO1, we incubated H4IIE cells for 4 h with vehicle (0.05% DMSO) or 10 µM of the proteasome inhibitor MG132 (upper panel). Cells were lysed, and equal protein amounts were used for Western blot analysis. Samples were probed for Nrf2 and NQO1, using actin as a loading control. Lower panel, densitometric analysis of Nrf2 (left) and NQO1 (right) protein normalized against β-actin. MG132 increased Nrf2 and NQO1 protein approximately two-fold. Graphs are representative of two independent experiments. (TIF) [file pone.0036774.s003.tif]

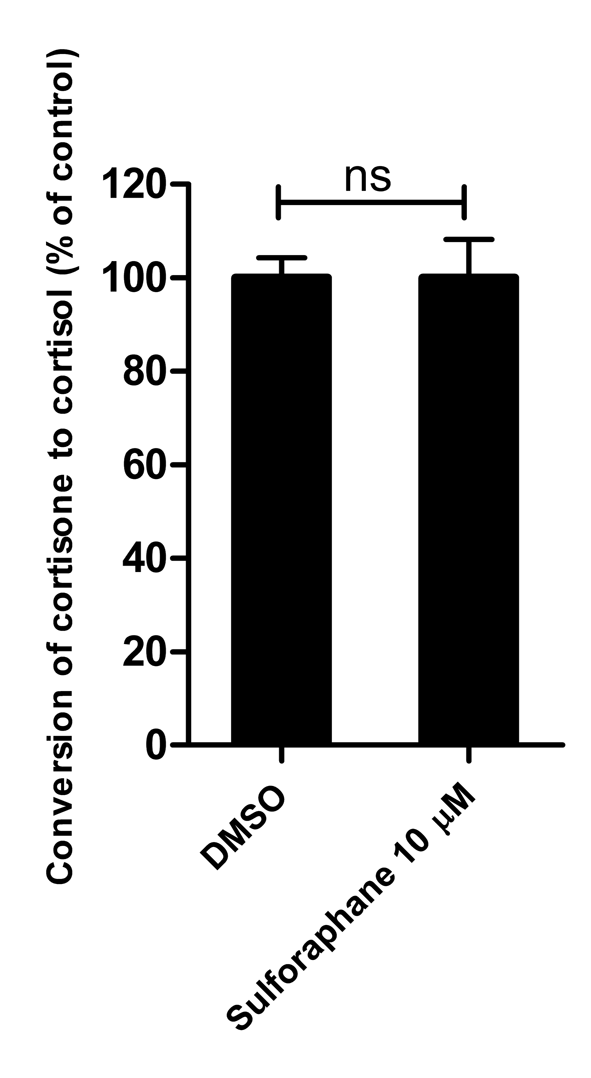

Supplement: Figure S2 — Sulforaphane does not inhibit 11β-HSD1 activity. Enzymatic activities were determined in intact H4H1 cells stably expressing 11β-HSD1. Briefly, 30,000 cells/well were seeded in 96-well plates (Becton-Dickinson, Basel, Switzerland). Cells were washed once 24 h later with 50 µL DMEMsf and incubated for another 3 h at 37°C. The medium was replaced by 40 µL fresh medium containing either vehicle or sulforaphane, and 10 µL medium containing 10 nCi [1,2-3H]-cortisone and 50 nM unlabeled cortisone to assess 11β-HSD1 reductase activity. Cells were incubated for 40 min at 37°C and reactions stopped by adding 2 mM of unlabeled cortisone and cortisol in methanol, followed by separation of steroids by thin layer chromatography and determination of the conversion of radiolabeled substrate by scintillation counting. 11β-HSD1 activity after sulforaphane treatment was indistinguishable from that of control cells treated with vehicle (DMSO). Data represent mean ± SD from at least three independent experiments measured in triplicate. P-value was determined using unpaired, two-tailed student t-test (ns, not significant). (TIF) [file pone.0036774.s004.tif]

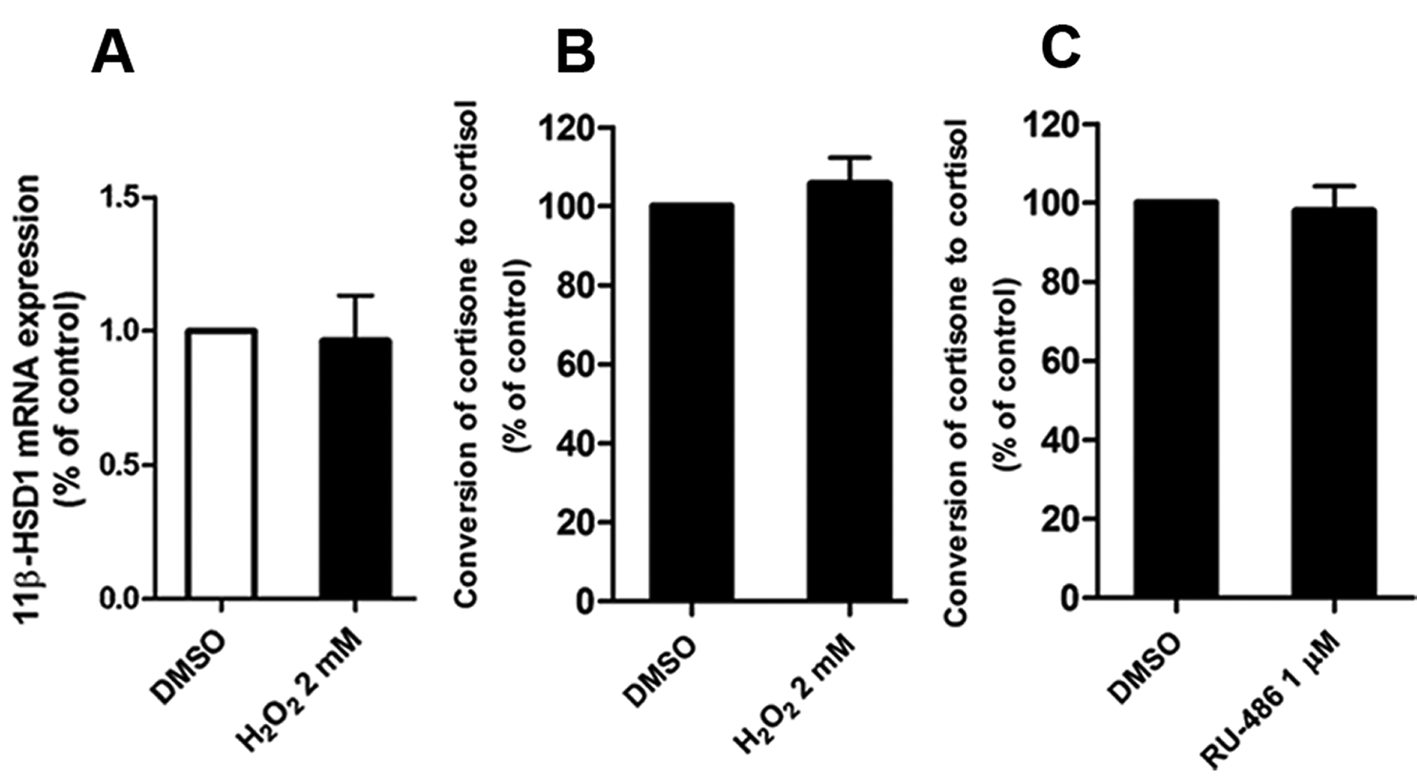

Supplement: Figure S3 — HSD11B1 mRNA expression and activity is unaffected by H2O2. To assess whether HSD11B1 mRNA expression is influenced by H2O2, HPCT-1E3 cells were cultivated for 24 h at 37°C in the presence of vehicle (0.05% DMSO) or H2O2 (2 mM) (A). Total mRNA was extracted using the Trizol method (Invitrogen, Carlsbad, CA). Total mRNA (2 µg) was reverse transcribed to cDNA using the Superscript-III First-Strand Synthesis System and oligo-dT (Invitrogen). Relative quantification of HSD11B1 mRNA expression was performed by RT-PCR on a RotorGene 6000 (Corbett, Australia) using the KAPA SYBR® FAST qPCR Kit (Kapasystems, Boston, MA). Relative HSD11B1 gene expression compared with the internal control GAPDH was determined using the delta-delta-CT method. Data (mean ± SD from two independent experiments measured in triplicate) represent ratios of 11ß-HSD1 mRNA to GAPDH control mRNA from treated cells normalized to the values obtained from cells incubated with vehicle (DMSO). Enzymatic activities were determined in intact HPTC-1E3 cells expressing endogenous 11β-HSD1 that were treated with vehicle (DMSO), 2 mM H2O2 (B) or 1 µM RU-486 (C). Briefly, 50'000 cells/well were seeded in 96-well plates (Becton-Dickinson, Basel, Switzerland). Cells were washed twice 12 h later with 50 µL DMEMct and incubated for another 24 h at 37°C. The medium was replaced by 40 µL fresh medium containing either vehicle, H2O2 (2 mM) or RU-486 (1 µM), and 10 µL medium containing 10 nCi [1,2-3H]-cortisone and 50 nM unlabeled cortisone to assess 11β-HSD1 reductase activity. Cells were incubated for 4 h at 37°C and reactions stopped by adding 2 mM of unlabeled cortisone and cortisol in methanol, followed by separation of steroids by thin layer chromatography and determination of the conversion of radiolabeled substrate by scintillation counting. Oxidative stress induced by H2O2 neither changed the expression of 11β-HSD1 mRNA nor its activity in HPCT-1E3 cells. Furthermore, the GR inhibitor RU-486 did not affect 11β-HS [file pone.0036774.s005.tif]

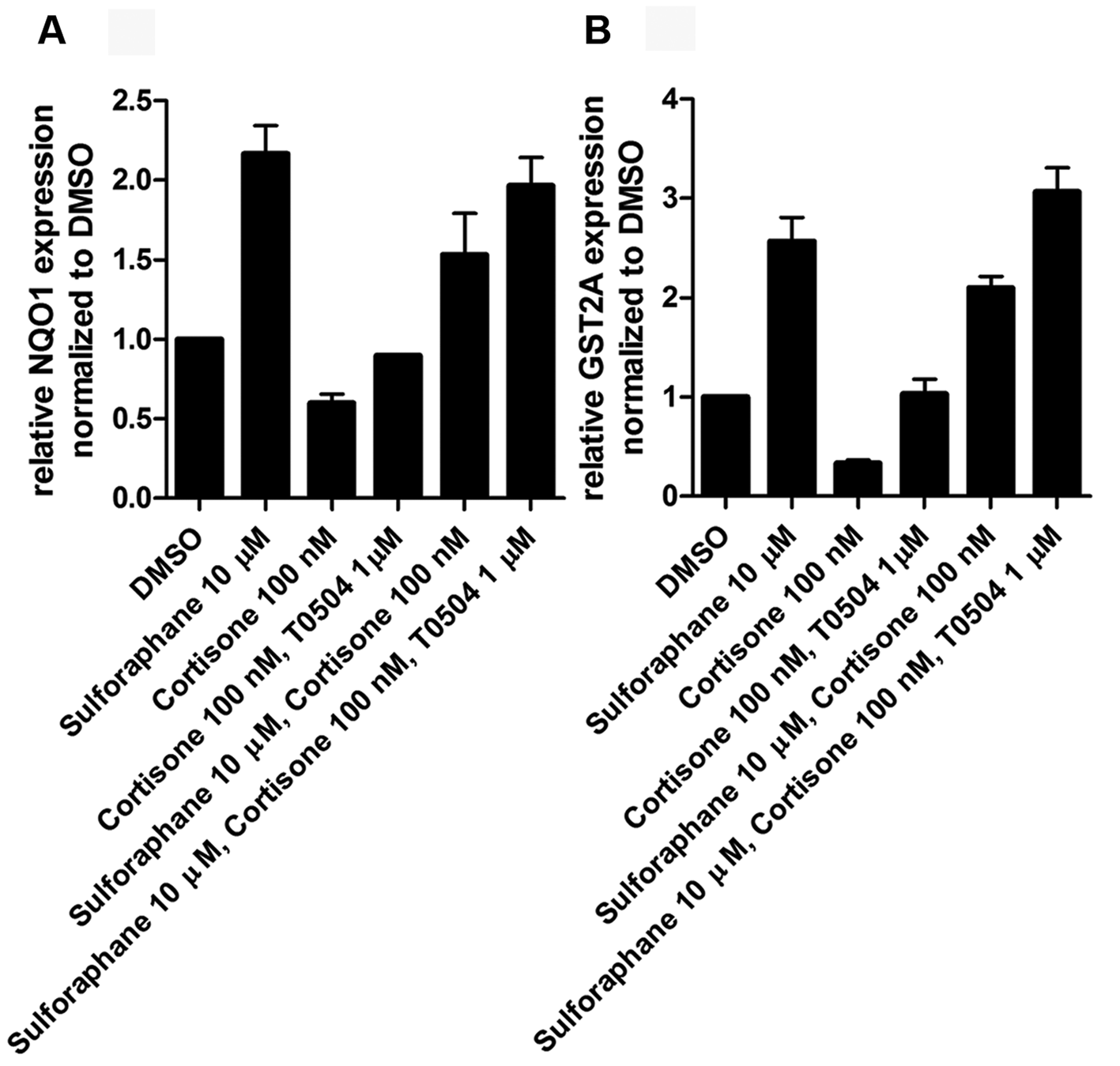

Supplement: Figure S4 — The 11β-HSD1 inhibitor T0504 restores NQO1 mRNA and GST2A mRNA expression in H4H1 cells. H4H1 cells (500'000 cells/well) were cultured in 24-well plates with DMEM for 12 h at 37°C. Cells were washed once with DMEMsf and incubated for another 3 h at 37°C. The culture medium was replaced with fresh DMEMsf containing sulforaphane (10 µM), T0504 (1 µM) or combinations of them, in the presence or absence of cortisone (100 nM), followed by incubation for 24 h at 37°C. Total mRNA was extracted using the Trizol method (Invitrogen, Carlsbad, CA). Total mRNA (2 µg) was reverse transcribed to cDNA using the Superscript-III First-Strand Synthesis System and oligo-dT (Invitrogen). Relative quantification of mRNA expression levels was performed by RT-PCR on a RotorGene 6000 (Corbett, Australia) using the KAPA SYBR® FAST qPCR Kit (Kapasystems, Boston, MA). Relative gene expression compared with the internal control GAPDH was determined using the delta-delta-CT method. Incubation with cortisone (100 nM) suppressed basal (0.5-fold and 0.3-fold) as well as sulforaphane-induced (1.5-fold and 2-fold) NQO1 (A) and GST2A mRNA (B) expression in H4H1 cells. Simultaneous treatment with the 11β-HSD1 inhibitor T0504 restored NQO1 and GST2A mRNA expression and fold activation was almost indistinguishable from cells treated with sulforaphane alone (2-fold and 2.8-fold) or vehicle control expression levels. Data represent mean ± SD from a representative experiment measured in triplicate. (TIF) [file pone.0036774.s006.tif]

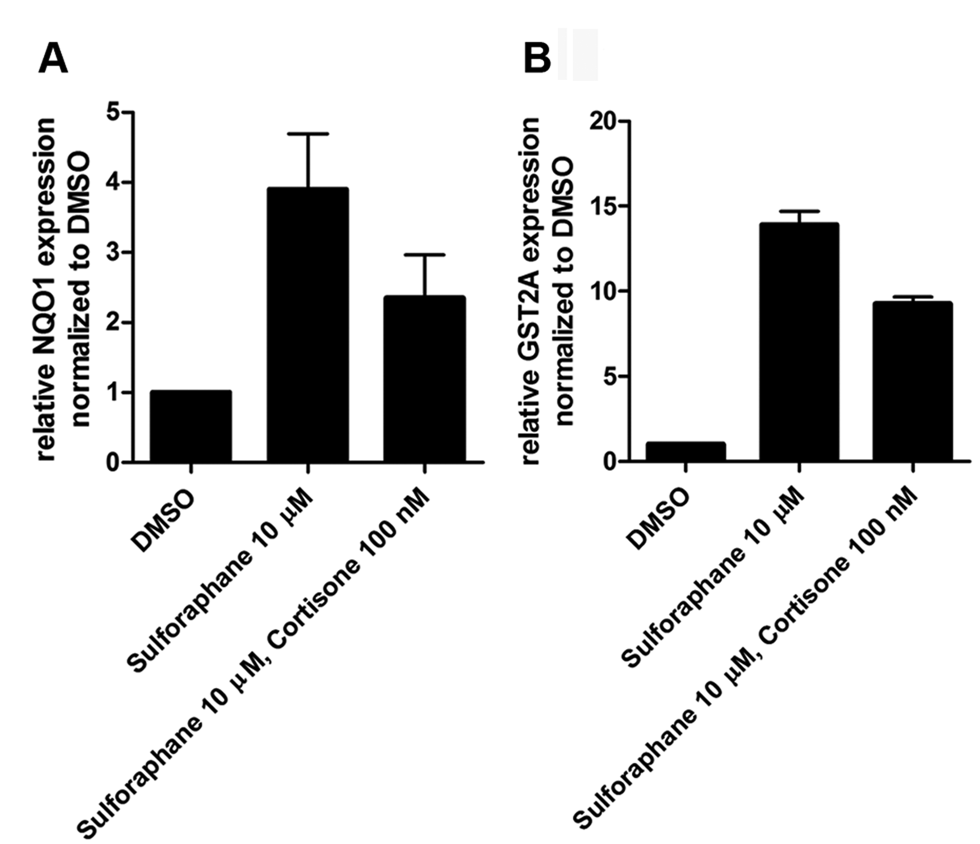

Supplement: Figure S5 — Incubation with cortisone suppresses NQO1 and GST2A mRNA expression in HPCT-1E3 cells with endogenous 11β-HSD1 activity. HPCT-1E3 cells (1'000'000 cells/well) were cultured in 6-well plates with dexamethasone supplemented DMEM for 12 h at 37°C. Cells were washed twice with DMEMct and incubated for another 3 h at 37°C. The culture medium was replaced with fresh DMEMct and cells were cultivated for another 24 h at 37°C. To assess whether basal 11β-HSD1 activity suppresses Nrf2, the culture medium was replaced with fresh DMEMct containing vehicle, sulforaphane (10 µM), T0504 (1 µM), and combinations of them, in the presence or absence of cortisone (100 nM) and cells were incubated for another 24 h at 37°C. Total mRNA was extracted using the Trizol method (Invitrogen, Carlsbad, CA). Total mRNA (2 µg) was reverse transcribed to cDNA using the Superscript-III First-Strand Synthesis System and oligo-dT (Invitrogen). Relative quantification of Nrf2 mRNA expression was performed by RT-PCR on a RotorGene 6000 (Corbett, Australia) using the KAPA SYBR® FAST qPCR Kit (Kapasystems, Boston, MA). Relative NQO1 mRNA and GST2A mRNA gene expression compared with the internal control GAPDH was determined using the delta-delta-CT method. NQO1 (A) and GST2A (B) mRNA expression levels were induced by sulforaphane (10 µM) 4-fold and 14-fold, respectively, in HPCT-1E3 cells. Cortisone (100 nM) treatment reduced both NQO1 and GST2A mRNA expression levels. Data represent mean ± SD from a representative (one out of three) experiment performed in triplicate. (TIF) [file pone.0036774.s007.tif]

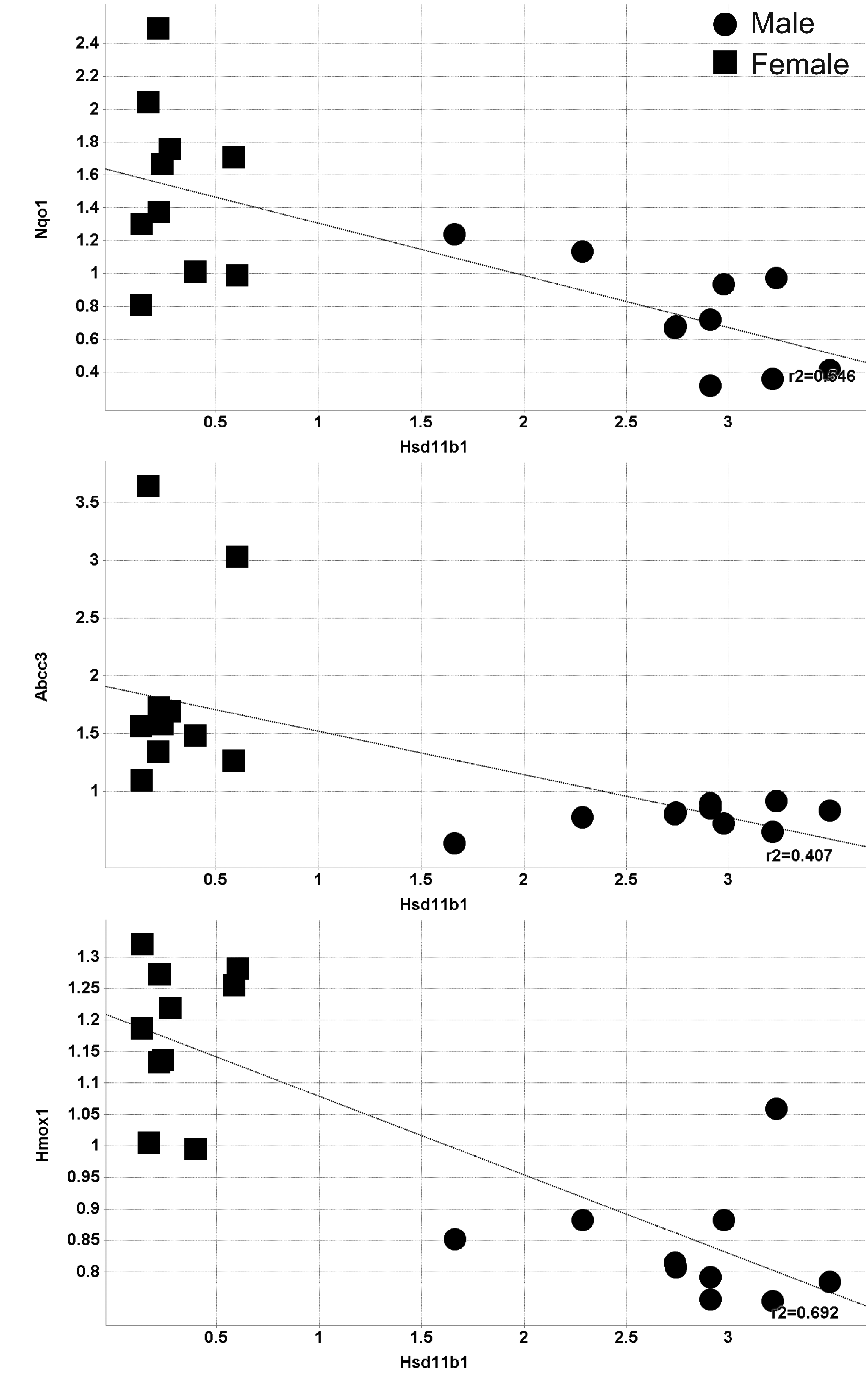

Supplement: Figure S6 — Expression of the Nrf2-dependent genes NQO1, Abcc3 and Hmox1 in comparison to HSD11B1 relative expression. HSD11B1 relative gene expression was evaluated in comparison to NQO1, Abcc3 and Hmox1, in each animal, by scatter plot. RNA purified from whole liver tissues of 20 Han Wistar rats (animals listed in Table S1) were hybridized to Rat Genome 230 2.0 chips (Affymetrix, Santa Clara, CA) for characterization of gene expression. Hybridization mixtures were prepared using the 3′-IVT Express Kit (Affymetrix) to accommodate 10 µg of labeled cDNA in 200 µL of hybridization mix. Rat Genome 230 2.0 Arrays were hybridized, revealed and washed according to the Affymetrix protocol. GeneChips were scanned using the Affymetrix 3000 scanner and images were converted to files (*.cel). The raw data were analyzed by GeneSpring GX 11.5.1 (Agilent) using the software default settings for Affymetrix expression chips (Table S2). Raw data were pre-processed by Robust Multi-array Analysis (RMA) algorithm (including Quantile normalization), Log transformed and then pre-filtered within the 20–100 percentile assuming the median as baseline (raw data). Of the 31099 probe-sets in Rat Genome 230 2.0 chip, 27359 have passed the pre-filter. Correlation plot (not shown) shows the correlation analysis (heat map, Pearson correlation factor) across arrays: high correlation degree is indicative of good experimental execution and high reproducibility. The internal quality controls of Affymetrix chips showed no abnormalities in the hybridization processes (not shown). Both analyses were part of the default quality controls. Linear regression analysis was used to obtain the overall correlation (R2) of HSD11B1 expression with other genes (HSD11B1 vs. Nqo1 R2 = 0.546, p value = 2.00E-004; HSD11B1 vs. Abcc3 R2 = 0.407, p value = 2.47E-003; HSD11B1 vs. Hmox1 R2 = 0.692, p value = 5.38E-006). The statistical relevance was assessed by multiple unpaired t-tests, with Benjamini Hochberg FDR multiple testing corr [file pone.0036774.s008.tif]
